# Label-Free Screening of Drug-Induced Liver Injury Using Stimulated Raman Scattering Microscopy and Spectral Phasor Analysis

**DOI:** 10.1021/acs.analchem.4c01285

**Published:** 2024-06-18

**Authors:** William J. Tipping, Liam T. Wilson, Nicholas C. O. Tomkinson, Karen Faulds, Duncan Graham

**Affiliations:** † Centre for Nanometrology, Department of Pure and Applied Chemistry, Technology and Innovation Centre, University of Strathclyde, Glasgow G1 1RD, U.K.; ‡ Department of Pure and Applied Chemistry, University of Strathclyde, Glasgow G1 1XL, U.K.

## Abstract

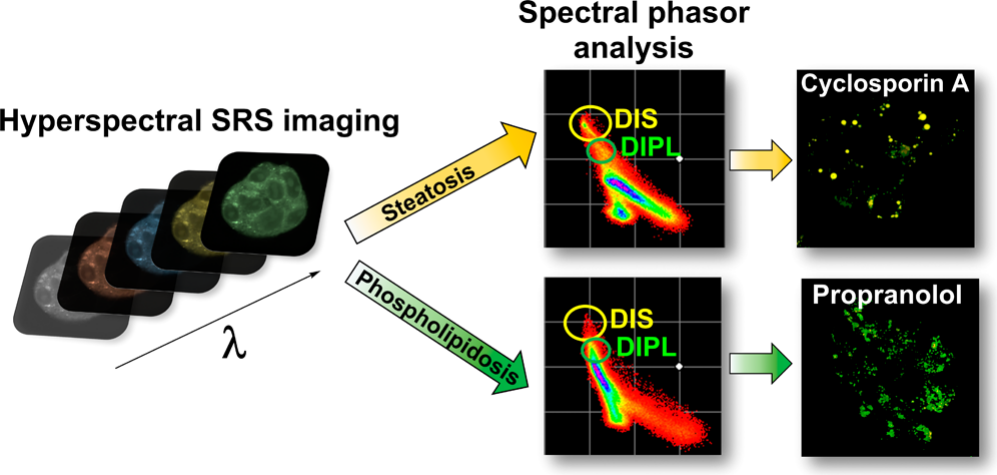

Hepatic toxicity is a leading cause of the termination of clinical trials and the withdrawal of therapeutics following regulatory approval. The detection of drug-induced liver injury (DILI) is therefore of importance to ensure patient safety and the effectiveness of novel small molecules and drugs. DILI encompasses drug-induced steatosis (DIS) and drug-induced phospholipidosis (DIPL) which involve the accumulation of excess intracellular lipids. Here, we develop hyperspectral stimulated Raman scattering (SRS) microscopy as a label-free methodology for discriminating DIS and DIPL in mammalian cell culture. We demonstrate that hyperspectral SRS imaging in tandem with spectral phasor analysis is capable of discriminating DIS and DIPL based on the nature and distribution of intracellular lipids resulting from each process. To demonstrate the practical application of this methodology, we develop a panel of alkyne-tagged propranolol analogues that display varying DILI effects. Using hyperspectral SRS imaging together with spectral phasor analysis, our label-free methodology corroborated the standard fluorescence-based assay for DILI. As a label-free screening method, it offers a convenient and expedient methodology for visualizing hepatotoxicity in cell cultures which could be integrated into the early stages of the drug development process for screening new chemical entities for DILI.

## Introduction

The liver plays a central role in the detoxification of small molecules and drugs, rendering it particularly susceptible to drug-induced liver injury (DILI).^1^ Hepatic toxicity has resulted in the termination of clinical trials and is the leading cause of the withdrawal of therapeutics following regulatory approval.^2^ DILI includes steatosis and phospholipidosis, which involve the production of cellular structures containing excess lipid. Drug-induced steatosis (DIS) refers to the accumulation of lipids in the form of diacyl and triacylglycerols together with cholesterol esters within intracellular lipid droplets (LDs), which are coated by a phospholipid monolayer. DIS is typically caused by an off-target effect of the drug, and although the precise mechanisms that drive DIS are not fully delineated, increased fatty acid uptake and drug inhibition of mitochondrial fatty acid oxidation are two proposed mechanisms, among others.^3^ Drug-induced phospholipidosis (DIPL) is the accumulation of phospholipid membranes within a multilayered/multilamellar structure.^4^ Cationic amphiphilic drugs (CADs) can instigate DIPL in cells and organs via accumulation in intracellular compartments, such as endosomes and lysosomes.^5^ Early detection of DIPL could mitigate these artifacts, enabling a focus on molecules with therapeutic potential in drug screening campaigns.^6^

A sequence of biochemical and morphological changes occurs following the exposure of cells to CADs. These include the formation of intracellular lipid droplets and the presence of lamellar bodies which incorporate drug and phospholipid species into concentric structures. The detection of DIPL using optical reagents has been previously reported, including the development of the commercial LipidTOX screening kit which contains two fluorescent stains: LipidTOX Red (for labeling phospholipids in DIPL) and LipidTOX Green (for labeling neutral lipid droplets resulting from DIS). The fluorescent reporters become incorporated into the lipid droplets and/or lamellar bodies that are produced in response to DIPL. As such, they inherently disrupt the biochemistry of each of these features. This issue is compounded by the requirement for LipidTOX Red as a cotreatment with the drug under investigation for the full duration of the drug treatment (typically >48 h). This requirement has driven the design of alternative fluorescent scaffolds for DIPL detection, including Nile Red,^7^ fluorescently labeled phospholipids,^8^ and two-photon fluorescent scaffolds.^9^ While these probes enable high-throughput fluorescence imaging that is well suited to early stage drug toxicity studies, off-target staining of the fluorophores and/or poor photostability cannot be overlooked, and in all cases, the hydrophobic labels directly impact the LD composition. Thus, label-free, noninvasive methods that can detect and differentiate DIPL and DIS in living cells would be highly advantageous to these current approaches.

Raman spectroscopy (RS) is a powerful technique for biomolecular characterization based on the inelastic scattering of incident light upon interaction with molecular vibrations. As a result, RS enables the characterization of endogenous cellular biomolecules, including lipids, proteins, and nucleic acids, in a label-free manner.^10^ Ratiometiric Raman imaging^11,12^ has been reported for visualizing the impact of drug treatment on cellular lipid metabolism.^13,14^ The development of stimulated Raman scattering (SRS) microscopy enables comparable imaging quality to confocal fluorescence microscopy, with rapid image acquisition rates, label-free detection, and subcellular spatial resolution (∼450 nm). Hyperspectral SRS imaging has become a high-content cellular imaging platform and the application of chemometric analysis techniques can extricate the underlying spectral changes associated with drug treatment or disease progression. In particular, multivariate curve resolution,^15^*k*-means cluster analysis^16,17^ and spectral phasor analysis^18−28^ have recently been applied to hyperspectral SRS imaging data for cellular and tissue studies.

Herein, we present the application of Raman spectroscopy and hyperspectral SRS imaging for the detection of DIPL and DIS in hepatocellular carcinoma cells. Initially, we focus on the application of hyperspectral SRS microscopy for detecting DIS and DIPL following treatment with drugs with known DILI activity. We demonstrated the application of spectral phasor analysis for the direct segmentation of intracellular lipid droplets during DIS and DIPL based directly on the corresponding SRS spectrum. Spectral phasor analysis enabled a direct differentiation between the two states of DILI. We then selected propranolol, which is known to result in DIPL, and prepared a series of alkyne-tagged derivatives in order to validate the use of the proposed method for identifying the features of DILI in live cell models with molecules of unknown DILI activity. Our method was shown to corroborate the LipidTOX assay which is the current gold-standard assay for the detection of DILI using optical reagents. This study highlights the potential of label-free Raman and SRS imaging for the detection of DIPL and DIS in mammalian cells that is ideally suited to high-throughput screens in the early stages of a drug discovery campaign.

## Results and Discussion

### SRS Microscopy of DIS and DIPL

**Figure 1:**
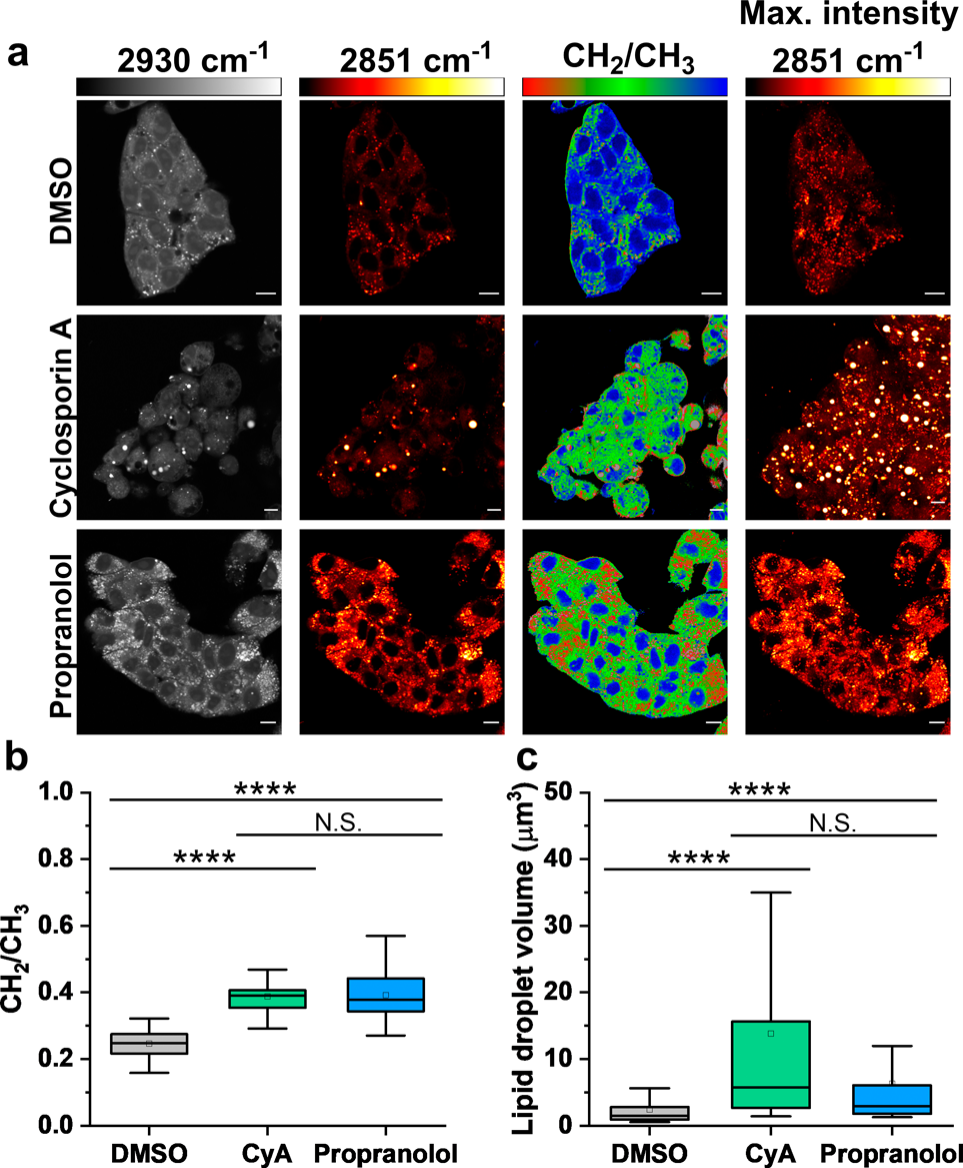
SRS imaging of drug-induced liver injury in live cells. HepG2 cells were treated with one of the following drugs: cyclosporin A (CyA, 20 μM) or propranolol (30 μM) for 48 h. (a) SRS images were acquired from live cell populations at 2930 cm^–1^ (CH_3_, proteins) and 2851 cm^–1^ (CH_2_ symmetric stretch). The ratio of the CH_2_/CH_3_ (2851 cm^–1^/2930 cm^–1^) is presented in Rainbow RGB LUT (0–1). The background (noncell areas) has been removed using an intensity threshold (see Experimental Section for details). A maximum intensity projection from a Z-stack of SRS images acquired at 2851 cm^–1^ (from the same cellular population) is also provided. Single-frequency SRS images were acquired at a frame size of 512 × 512 pixels, using a 48 μs pixel dwell time with false colors applied to different detection wavenumbers. Scale bars: 10 μm. (b) Average live single-cell CH_2_/CH_3_ ratios from the SRS images for each condition (*n* > 30 cells per condition over three independent experiments). (c) Quantification of the lipid droplet volume determined from the image Z-stacks acquired at each respective treatment condition (*n* > 30 cells per condition over three independent experiments). In panels b and c, data are plotted as boxplots: center line indicates median; box limits indicate upper and lower quartiles; whiskers indicate minimum and maximum. Statistical significance was determined using a one-way ANOVA with a posthoc Tukey test; N.S. not significant, *** *P* ≤ 0.001.

SRS microscopy enables fast image acquisition, subcellular resolution, and linear concentration dependence for straightforward interpretation of cellular components. We therefore explored the use of SRS microscopy to visualize the phenotypic outcomes of DIS and DIPL in live HepG2 cells, which are routinely used as a model cell line for DILI investigations.^29^ Live HepG2 cells were treated with cyclosporin A, which is known to result in DIS;^9^ propranolol, which is known to result in DIPL; or DMSO as a control (Figure 1a).^30^ SRS images were acquired by tuning the frequency difference between the pump and Stokes beams to be resonant with intracellular protein (2930 cm^–1^, CH_3_ symmetric stretch) and lipid species (2851 cm^–1^, CH_2_ symmetric stretch).^31,32^ The image acquired at 2930 cm^–1^ highlights the protein signal throughout the cell cytoplasm, cell nuclei, and nucleoli in each treatment condition. The cellular lipid pool is clearly visualized using the 2851 cm^–1^ signal, and the detection of cellular lipid droplets is prominent in these images. Ratiometric analysis of the CH_2_/CH_3_ resolved the lipid droplets with a relatively high ratio value (>0.8), while the nuclear regions generated a relatively low ratio value (<0.2) reflecting the low lipid content within the nuclear compartment. We quantified the CH_2_/CH_3_ ratio in the cells under all treatment conditions which revealed that, compared to the DMSO control, both drug treatments increased the CH_2_/CH_3_ ratio (Figure 1b). Furthermore, to visualize the LD pool across the whole cell volume, three-dimensional (3D) imaging was performed at 2851 cm^–1^ by adjusting the z-focal plane in between image frames (*z* = 1 μm). A maximum intensity projection was created which rendered the 3D data set into a 2D image based on the maximum voxel intensity. The drug treatments resulted in an increased lipid signal throughout the 3D volume compared to the DMSO control. We quantified the lipid droplet volume under each condition which revealed that cyclosporin A treatment had resulted in the formation of large lipid droplets relative to the control sample (Figure 1c). This result is consistent with the detection of DIS where the accumulation of lipids into lipid droplets is known to occur. Interestingly, cyclosporin A treatment resulted in a significant increase in LD volume relative to the control, whereas propranolol **1** did not, while a significant difference in the CH_2_/CH_3_ ratio was detected for both drugs relative to the DMSO control. These results suggested that the detection of DIS and/or DIPL cannot be determined purely on the LD volume or the CH_2_/CH_3_ ratio alone.

To improve the reliability of LD segmentation, we elected to use spectral phasor analysis for cellular segmentation based directly on the SRS spectrum. Spectral phasor analysis uses a Fourier transform to project the spectrum of every pixel in the image stack as a point on the phasor plot.^18^ The spectral phasor therefore represents a density plot of the ensemble pixels which are clustered based on spectral similarity, thereby simplifying the interpretation and segmentation of hyperspectral imaging data sets. Previous studies have highlighted the application of spectral phasor analysis for visualizing the phenotypic outcomes of drug treatment,^21,26,28^ imaging ratiometric alkyne sensors,^22^ and for investigating cellular mitosis.^28^ In each of these studies, the biomolecular content of each cluster in the spectral phasor plot was projected back onto the original data set. As such, the primary application of spectral phasor analysis has been for label-free organelle detection when using hyperspectral SRS imaging. We therefore set out to determine the potential of spectral phasor analysis to identify spectral features of lipid droplets during DIS and DIPL, using the phasor plot as a convenient method to compare hyperspectral SRS data sets directly. We selected the fatty acids arachidic acid, oleic acid, linolenic acid, and arachidonic acid to investigate the lipid unsaturation in the phasor domain. In addition, we investigated the membrane lipids, L-α-phosphatidyl choline, sphingomyelin, glyceryl tristearate, cholesterol, and cholesterol lineolate which are reflective of the lipid composition in mammalian cell systems.^33^

**Figure 2:**
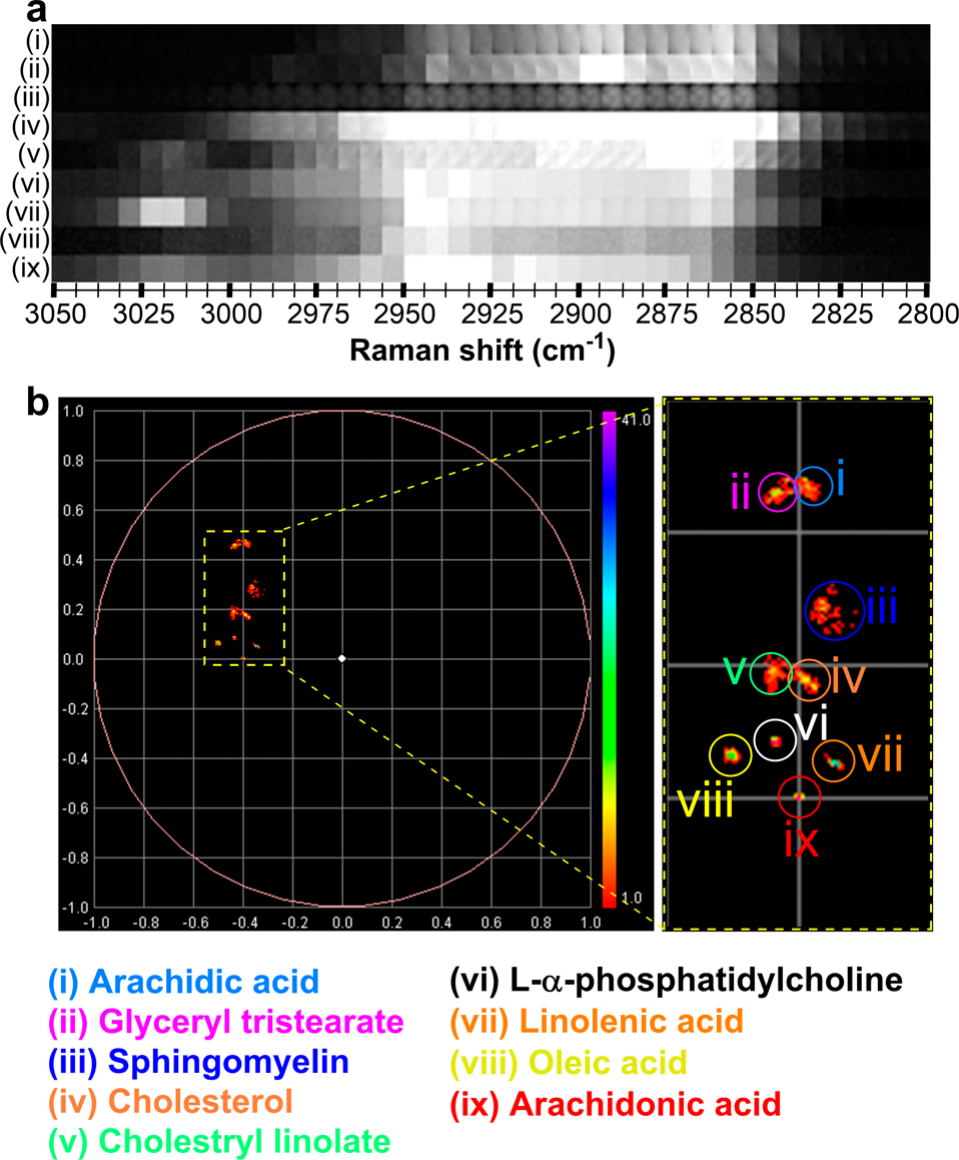
Hyperspectral SRS imaging of neat lipids. (a) SRS images were acquired across the range 3050–2800 cm^–1^ (using a 0.4 nm retuning of the pump laser in between image frames totalling 40 images) of neat lipid species. Each frame is 5.7 μm^2^: (i) arachidic acid, (ii) glyceryl tristearate, (iii) sphingomyelin, (iv) cholesterol, (v) cholesteryl lineolate, (vi) L-α-phosphatidylcholine, (vii) linolenic acid, (viii) oleic acid, and (ix) arachidonic acid. (b) Spectral phasor analysis of the hyperspectral SRS image stacks acquired in panel a.

Hyperspectral SRS images were acquired from a series of neat lipid samples by sequential retuning of the pump laser wavelength in increments of 0.4 nm to create a stack of images covering the range 2800–3050 cm^–1^ of the Raman spectrum (Figure 2a). We applied spectral phasor analysis from a combined image stack of the nine lipid species, which showed each lipid occupied a discrete region in the phasor plot (Figure 2b). When comparing the fatty acids species (i, vii, viii, and ix), the clustering of the phasors is closer to the origin of the phasor plot with an increasing number of unsaturated C=C in the lipid tail. For example, saturated lipids including arachidic acid (i) and glyceryl tristearate (ii) were clustered furthest away from the origin of the phasor plot, while unsaturated fatty acids, linolenic acid (vii), and arachidonic acid (ix) were clustered closer to the phasor origin. Meanwhile, cholesterol esterification was effectively identified within the phasor domain (cholesterol (iv) vs. cholesteryl lineolate (v)), with a clear peak at ∼3015 cm^–1^ indicative of the unsaturated lineolate tail (Figure 2a). Lastly, the membrane lipid, sphingomyelin (iii) and the phospholipid, L-α-phosphatidyl choline (vi) were well resolved from each other in the phasor plot. Altogether, these results demonstrated the power of spectral phasor analysis for discriminating different lipid species based directly on the hyperspectral SRS spectrum.

**Figure 3:**
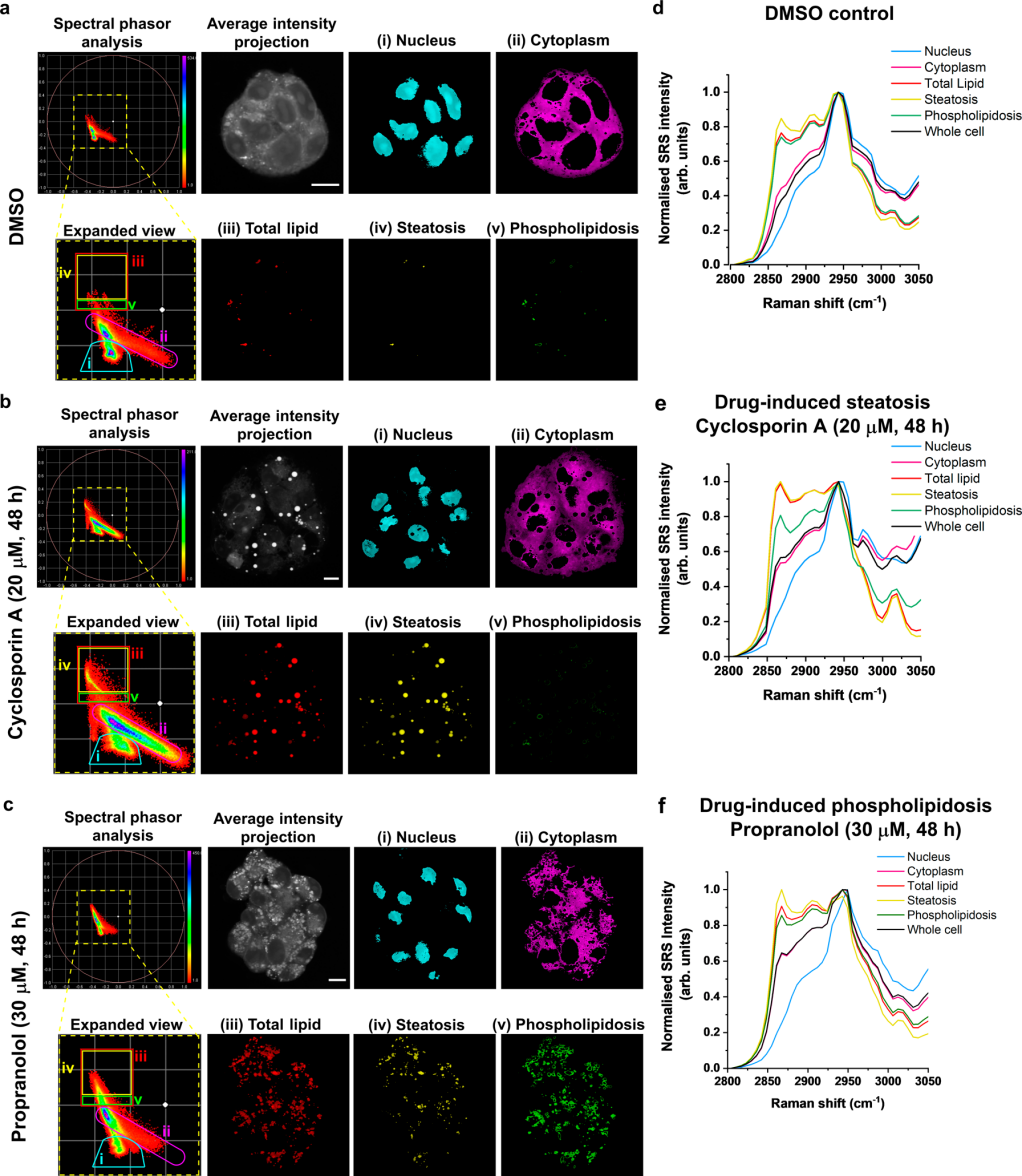
Investigating drug-induced liver injury using hyperspectral SRS microscopy and spectral phasor analysis. HepG2 cells were treated with (a) DMSO (control), (b) cyclosporin A (20 μM, 48 h), or (c) propranolol (30 μM, 48 h) before fixing (using 4% paraformaldehyde in PBS). Hyperspectral SRS imaging was performed across the range 2800–3050 cm^–1^ (0.4 nm retune, 40 images) from a minimum of three biological replicates. A spectral phasor analysis of the hyperspectral SRS image stack was performed. The yellow dashed marker represents the area of the spectral phasor plot that is selected in the expanded view, which has been segmented based on the color-coded markers into (i) nucleus (cyan), (ii) cytoplasm (magenta), (iii) total lipid (red), (iv) steatosis (yellow), and (v) phospholipidosis (green). An average intensity projection is also provided (scale bars: 10 μm). (d–f) Average normalized SRS spectra corresponding to the ROIs selected in panels a–c.

Having demonstrated that spectral phasor analysis can differentiate the lipid species tested, we next investigated if hyperspectral SRS imaging and spectral phasor analysis could discriminate DIS and DIPL in cell culture, given that the two processes result in the accumulation of different lipid species.^34^ First, HepG2 cells were treated with DMSO (Figure 3a), cyclosporin A (20 μM, 48 h) (Figure 3b), or propranolol (30 μM, 48 h) (Figure 3c). In each case, a hyperspectral SRS image stack was acquired across the range of 2800–3050 cm^–1^ from which a spectral phasor analysis and average intensity projection were created. In addition, the normalized SRS spectra from the segmented regions of the spectral phasor plots are presented for each treatment condition in Figure 3d–f. Based on our previous reports,^21−23^ the cellular nuclei (i, cyan) and cytoplasm (ii, magenta) were easily identified based on their relative positions on the phasor plot and the associated SRS spectra. For example, the nuclear SRS spectrum presented a peak at 2965 cm^–1^ indicative of cellular DNA.^35^ We next selected the area in (iii), red designated “total lipid” given the relative clustering of the nine lipid species in the spectral phasor plot of the neat samples presented in Figure 2. Noticeably, the segmented images of the region (iii) total lipid showed that the treatment of HepG2 cells with cyclosporin A or propranolol resulted in significant lipid accumulation relative to the DMSO control. In addition, the normalized SRS spectra associated with the HepG2 cells treated with cyclosporin A (Figure 3e) and propranolol (Figure 3f), and hence indicating DILI, show an increased relative intensity of the CH_2_ symmetric stretch ∼2850 cm^–1^ compared to the DMSO control (Figure 3d). Together these data confirm that DILI manifests as increased lipid content in HepG2 cells when treated with either cyclosporin A or propranolol.

We investigated the segmentation of the region of the spectral phasor plot (iii) total lipid further (Figure 3a–c). In the cyclosporin A-treated population, there were a large number of spectral phasors which were identified in the yellow ROI which we have ascribed as (iv) steatosis, reflecting the known activity of cyclosporin A to induce DIS. Conversely, in the propranolol-treated cells (Figure 3c), the phasors were largely confined to the region identified in the green ROI which were termed (v) phospholipidosis, reflecting the known potency of propranolol toward DIPL. In each case, the segmentation of the lipid region of the spectral phasor plot indicated that cyclosporin A treatment resulted in large lipid droplets which were mostly detected in the yellow ROI of the phasor plot ((iv) steatosis), with a negligible signal detected in the green ROI ((v) phospholipidosis). Conversely, in the propranolol-treated cells, the predominant clustering of the phasors was detected in the green ROI ((v) phospholipidosis). The normalized SRS spectra indicated the chemical differences between the ROIs in the lipid region of the spectral phasor plot. First, cyclosporin A treatment showed that the (iv) steatosis ROI resulted in a large peak ∼3010 cm^–1^ (=CH) indicative of unsaturated lipid and triacylglycerol content relative to the average spectra of the yellow ROI in the DMSO control and propranolol-treated cells. Meanwhile, the green ROI (v) phospholipidosis represented the area of the spectral phasor plot with the majority of the phasors detected in the propranolol-treated cells (Figure 3c).

**Figure 4:**
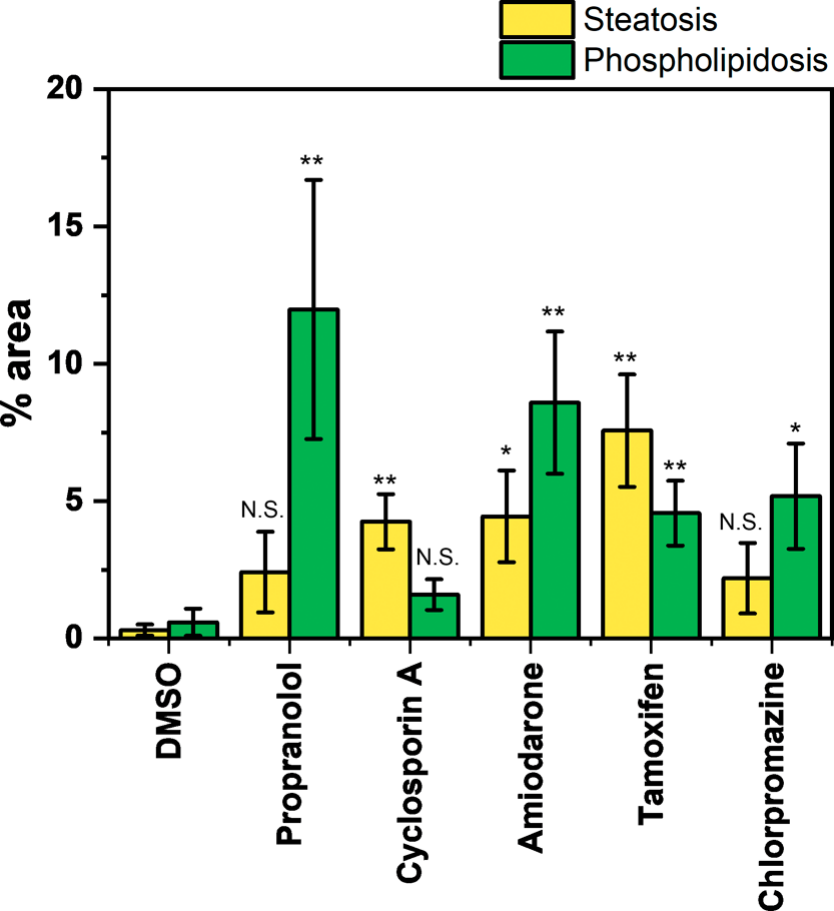
Analysis of drug-induced effects using hyperspectral SRS imaging. The percentage area of lipid signals of the segments ascribed to (iv) steatosis and (v) phospholipidosis detected in HepG2 cells following the respective drug treatments in Figure 3 and Figure S1. Data represent the mean percentage area of each segment across a minimum of five replicate images for each drug treatment, error bars: ± SD. Statistical significance was determined using a student’s *t* test; N.S. not significant, * *P* ≤ 0.05, ** *P* ≤ 0.01.

Having demonstrated that the two subsets of DILI result in different clustering patterns in the spectral phasor analysis, we supported these results with additional treatments with drugs of known DILI activity. HepG2 cells were treated with amiodarone (10 μM, 48 h), tamoxifen (10 μM, 48 h), or chlorpromazine (10 μM, 48 h) prior to hyperspectral SRS imaging as described previously. We performed the spectral phasor analysis and segmentation of the total lipid region into the classifications for steatosis and phospholipidosis (Figure S1). Our results demonstrated that each drug resulted in an increase in the lipid content relative to the DMSO control population. We also quantified the % area of the lipid droplets resulting from the yellow ROI, (iv) steatosis, and the green ROI, (v) phospholipidosis, for each drug treatment (Figure 4). Our results indicated that the predominant lipid signal for propranolol corresponds to phospholipidosis, which showed a significant increase in % area relative to the control, whereas in the case of cyclosporin A, steatosis was shown to represent the major lipid accumulation type (Figure 4). Amiodarone and tamoxifen were shown to result in significant lipid accumulation with a significant % area of both steatosis and phospholipidosis being detected under these treatments. These observations are in agreement with a previous report for DILI detection using an assay based two-photon fluorescence.^9^ Lastly, chlorpromazine was shown to result in phospholipidosis, a result which has been recently reported as the underlying mechanism identified in drug screening trials against SARS-CoV-2 by which chlorpromazine exerts a therapeutic effect.^36^ Together these data indicated that using hyperspectral SRS imaging with spectral phasor analysis, the detection of DILI, and subsequent classification into steatosis and/or phospholipidosis was possible with good agreement with previous imaging-based assays and clinical observations. In addition, this study highlights the power of SRS imaging, harnessing the high spatial resolution for suborganelle visualization, together with hyperspectral imaging for understanding the molecular composition across the lipid droplet without the use of hydrophobic markers and stains to do so.

### Detecting DILI Across a Panel of Novel Small Molecules

**Figure 5:**
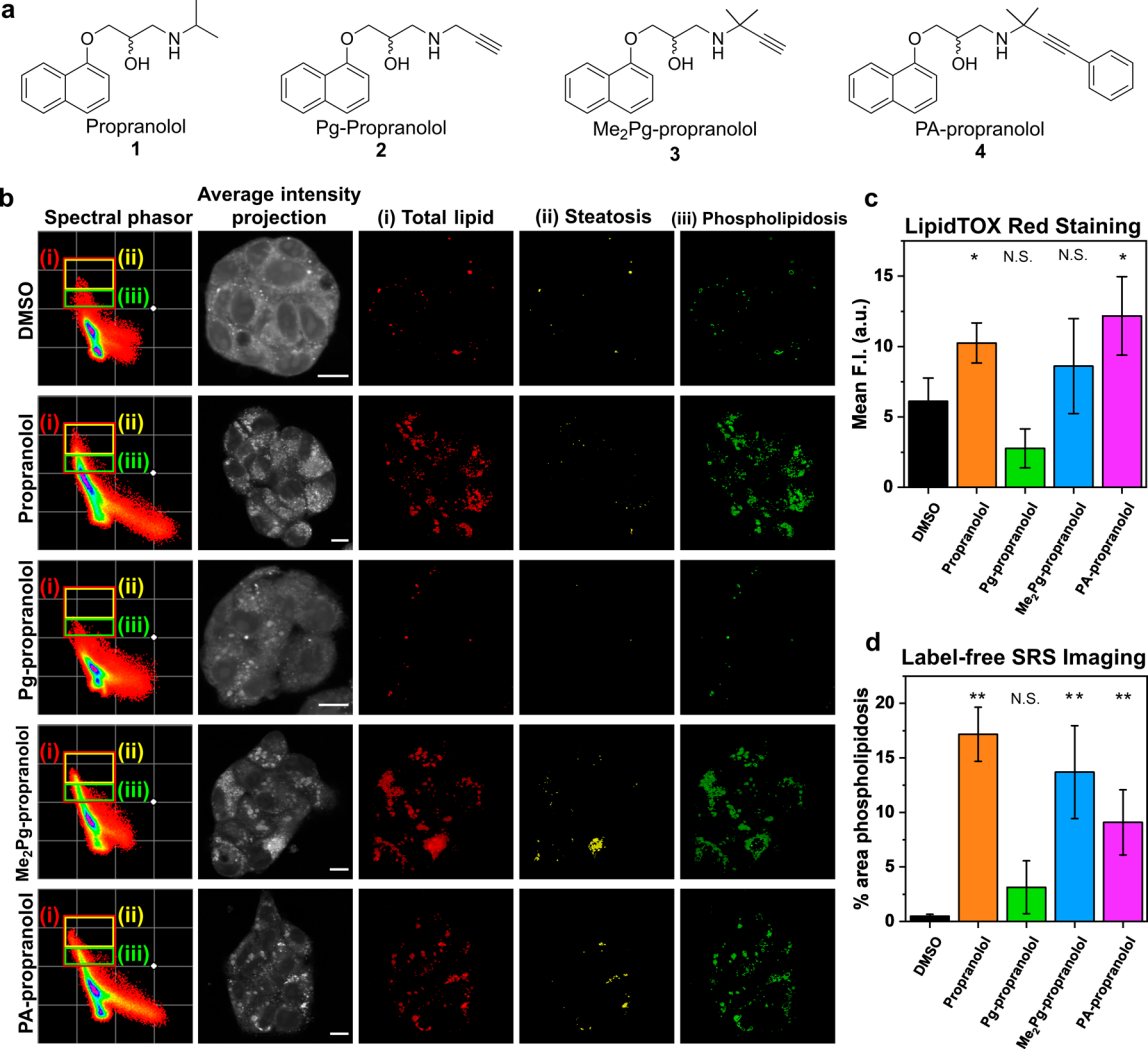
Hyperspectral SRS imaging and spectral phasor analysis of drug-induced phospholipidosis (DIPL). (a) Chemical structures of propranolol (**1**) and propranolol analogues **2**–**4**. (b) HepG2 cells were treated with DMSO (control), propranolol (30 μM, 48 h) or propranolol analogue **2**–**4** (30 μM, 48 h) before hyperspectral SRS images were acquired across the range 2800–3050 cm^–1^ (0.4 nm retune, 40 images). A spectral phasor analysis of the hsSRS image stack is provided. An expanded view of the spectral phasor plot has been segmented based on the color-coded markers into (i) total lipids, (ii) steatosis, and (iii) phospholipidosis. An average intensity projection is also provided (scale bar: 10 μm). (c) Detection of DIPL via LipidTOX Red staining. Mean fluorescence intensity (F.I.) at each treatment condition determined per cell image (>10 cells per image, min. Three replicate images). Data represent mean F.I. error bars ± SD. (d) Analysis of the percentage cell area phospholipidosis (iii, green) per cell area. Data represent the mean % area from >3 biological replicates with error bars: ± SD. Statistical significance was performed using a student’s *t* test to compare the drug treatments relative to the DMSO control; * *P* ≤ 0.05, ** *P* ≤ 0.01.

Having demonstrated the use of hyperspectral SRS imaging with spectral phasor analysis for discriminating DIS and DIPL in cell culture using drugs with known DILI behavior, we investigated whether the approach was successful for molecules with unknown DIPL activity. We therefore selected propranolol **1**, which is known to induce DIPL in cell cultures,^37^ to be used as a model compound for investigating DIPL using a Raman imaging-based approach. The development of an alkyne-labeled propranolol analogue would enable further investigation of the development of DIPL at the cellular level using alkyne-tag Raman imaging. The structure–activity relationship of propranolol **1** has been extensively studied,^38^ where bulky functionalization of the secondary amine generally conserves the adrenergic activity (Figure S2a). We therefore synthesized three alkyne labeled propranolol analogues (**2**–**4**) as potential mimics for the intracellular activity of propranolol (Figure 5a). The Raman spectra of each of the compounds show a discrete peak in the cell-silent region, and when normalized to the intensity of the naphthalene CH bending mode at 1575 cm^–1^, the relative intensities of the alkyne groups can be compared (Figure S2b). Conjugation of the alkyne to an aromatic ring, such as in PA-propranolol **4**, generated the greatest Raman scattering intensity (Figure S2c), which is consistent with previous studies of the structure-Raman shift/intensity of alkyne groups.^39^ Additionally, it increases the molecular weight and overall cLogP of compound **4**, which may increase the propensity of PA-propranolol toward DIPL induction when compared to the terminal alkyne compounds **2** and **3**, respectively. Finally, PA-propranolol **4** can be detected within the low mM range in solution using Raman spectroscopy for high-sensitivity detection (Figure S 2d). The physiochemical properties, cLogP and p*K*_a_, of the trio of compounds (**2**–**4**) were evaluated against a set of drugs with known positive/negative DIPL activity (Figure S3). Perhaps unsurprisingly, the increased hydrophobicity of PA-propranolol **4** indicated a higher propensity for DIPL induction, while removal of the geminal methyl groups in **2** effectively predicts little/no DIPL activity. However, the DIPL activity of **3** is less apparent; DIPL activity is not reliably predicted where scores are in the range 85 > [p*K*_a_]^2^ + [cLogP]^2^ > 75.^40^ This observation reinforces the need for an analytical approach to assess novel compounds for potential DIPL.

Spectral phasor analysis was performed on HepG2 cells treated with propranolol, an alkyne-labeled propranolol analogue (**2**–**4**), or DMSO as a control. HepG2 cells treated with propranolol resulted in significant number of phasors in (i) total lipid (red marker) and (iii) phospholipidosis (green marker); signals indicative of DIPL (Figure 5b). This is consistent with the known DIPL activity associated with propranolol and was validated by positive LipidTOX Red staining which is the current gold-standard optical reagent for visualizing DIPL (Figure S4 and Figure 5c). Interestingly, the two alkyne-labeled analogues with high LogP values, Me_2_Pg-propranolol **3** and PA-propranolol **4**, resulted in similar phospholipid accumulation; while PA-propranolol **4** resulted in an increased LipidTOX red staining, Me_2_Pg-propranolol **3** did not (Figure 5c). We quantified the % area of the signal in (iii) phospholipidosis using label-free SRS imaging and spectral phasor analysis, which showed that across three biological replicates, propranolol **1**, Me_2_Pg-propranolol **3**, and PA-propranolol **4** resulted in phospholipidosis relative to the DMSO control, whereas Pg-propranolol **2**, which had the lowest LogP value, did not result in DIPL (Figure 5d). We also investigated the impact of the propranolol analogues (**2**–**4**) using ratiometric Raman imaging (Figure S5), which demonstrated that an increased lipid content was detected in cells treated with Me_2_Pg-propranolol **3** and PA-propranolol **4**, but not Pg-propranolol **2**. These data provide an insight into the structure–DIPL activity of a small series of novel propranolol analogues which had unknown DILI activity. Structure–activity relationship (SAR) profiling as a predictor of phospholipidosis has been attempted by multiple *in silico* and *in vitro* methods, although major considerations remain;^41^ for example, the use of chemical labeling agents directly affecting the metabolism of potential CADs in drug screening assays. The present study highlights the benefit of a label-free SRS microscopy approach for visualizing DIPL susceptibility in the early stages of drug design and development campaigns. Together, these data indicate the potential for discriminating DIS and DIPL in cell cultures in a label-free way using spectral phasor analysis. This is a particular advantage compared to the use of the fluorescent stains, LipidTOX Green (for staining DIS) and LipidTOX Red (for staining DIPL), which colocalize within cells thereby complicating the independent detection of DIS and DIPL (Figure S6).

Given that PA-propranolol resulted in significant DIPL activity, we therefore used alkyne-tag Raman imaging^42^ to understand the intracellular localization of CADs. In HepG2 cells treated with PA-propranolol, we observed colocalization of the total lipid signal at 2851 cm^–1^ and the alkyne signal of PA-propranolol at 2233 cm^–1^ using SRS microscopy (Figure S7), together with colocalization of PA-propranolol with cellular phospholipid signal at 736 cm^–1^ in the Raman image (Figure S5c). Our data indicated that PA-propranolol was likely to be internalized into concentric lamellar structures typically associated with DIPL, thus representing the likely mechanism of disease progression. Furthermore, this result supports the conclusion that the region of the green segment (labeled phospholipidosis) is a result of DIPL and that the lamellar bodies are the likely origin of this signal identified within the phasor plot. Going beyond the current study, it would be of interest to probe the metabolism of PA-propranolol during DIPL in HepG2 cells, as a previous mass spectrometry-based analysis revealed lipidated homologues of propranolol have been detected and could be used as further indicators of the propensity toward phospholipidosis.^43^ In addition, a recent study developed a Penalized Reference Matching algorithm for SRS microscopy as a label-free means to identify the composition of lipid droplets in a variety of models.^44^ Together, the high spatial resolution and molecular specificity afforded by SRS microscopy could reveal novel insights into phospholipidosis at the molecular scale.

## Conclusions

We have demonstrated the application of hyperspectral SRS imaging coupled to spectral phasor analysis for the discrimination of DIS and DIPL in mammalian cell cultures. Spectral phasor analysis was able to discriminate nine lipid species, and the compositional shift of lipid accumulation during DIS and DIPL was also investigated. Spectral phasor analysis showed that DIS resulted in large lipid droplet formation and also represented a convenient method to directly compare the DIPL effects in a test series of alkyne labeled propranolol molecules. The results of label-free SRS imaging were validated by multimodal imaging with LipidTOX Red as an established optical method for DIPL imaging. Our method for discriminating DIS and DIPL in cell cultures outperformed the conventional LipidTOX screening method because it is label-free and therefore is not at risk of causing off-target staining. Furthermore, using the alkyne group present in PA-propranolol, we visualized the accumulation of the molecule in lipid-rich inclusions, supporting the hypothesis that CADs become trapped within lysosomes bound to lamellar phospholipid-rich bodies. We propose that by taking advantage of the biocompatibility of SRS imaging together with perfusion chamber cell culture,^45^ the progression of DIPL in live cells could be investigated using PA-propranolol to probe lamellar body formation during CAD exposure. In addition, the application of machine learning to spectral phasor segmentation could improve the compatibility of the technique for high-throughput screening in the early stages of drug development. Notwithstanding the observation that DILI is a major cause of the withdrawal of approved drugs, the development of label-free SRS imaging for DIS and DIPL could reduce the risk of hepatotoxicity in the earlier stages of drug development campaigns with clear and obvious financial and patient safety benefits.

## Experimental Section

### Reagents and Chemicals

(±)-Propranolol hydrochloride was purchased from Sigma-Aldrich and used as supplied. The alkyne-labeled analogues (**2**–**4**) were synthesized in house (see the Supporting Information). Stock solutions of each compound were prepared at a concentration of 100 mM in anhydrous DMSO. Amiodarone, chlorpromazine, cyclosporin A, and tamoxifen were all purchased from Sigma-Aldrich and used as supplied. Stock solutions were prepared at 50 mM in anhydrous DMSO.

### Cell Culture

HepG2 cells were purchased as an authenticated stock from the European Collection of Authenticated Cell Cultures (ECACC) operated by Public Health England (catalogue number 85011430). HepG2 cells were cultured in Dulbecco’s modified Eagle medium low glucose (DMEM containing 1 g/L glucose, GIBCO, Fisher Scientific) supplemented with 10% fetal bovine serum (FBS, Gibco, Fisher Scientific), 1% penicillin/streptomycin (Gibco, 10 000 U mL^–1^, Fisher Scientific), and 1% amphotericin B (Gibco, 250 μg mL^–1^, Fisher Scientific). Cells were maintained at 37 °C and 5% CO_2_ in a humidified incubator and were routinely subcultured at *ca*. 80% confluency.

### SRS Microscopy

An integrated laser system (picoEmerald S, Applied Physics & Electronics, Inc.) was used to produce two synchronized laser beams at 80 MHz repetition rate. A fundamental Stokes beam (1031.4 nm, 2 ps pulse width) was intensity modulated by an electro-optic-modulator (EoM) with >90% modulation depth, and a tunable pump beam (700–960 nm, 2 ps pulse width, < 1 nm (<10 cm^–1^) spectral bandwidth) was produced by a built-in optical parametric oscillator. The pump and Stokes beams were spatially and temporally overlapped using two dichroic mirrors and a delay stage inside the laser system and coupled into an inverted laser-scanning microscope (Leica TCS SP8, Leica Microsystems) with optimized near-IR throughput. SRS images were acquired using a 40× objective (HC PL IRAPO 40×, N.A. 1.10 water immersion lens) with a 9.75–48 μs pixel dwell time over a 512 × 512 or a 1024 × 1024 frame. The Stokes beam was modulated with a 20 MHz EoM. Forward scattered light was collected by a S1 N.A. 1.4 condenser lens (Leica Microsystems). Images were acquired at 12-bit image depth. The laser powers measured after the objective lens were in the range of 10–30 mW for the pump beam only, 10–50 mW for the Stokes beam only, and 20–70 mW (pump and Stokes beams). The spatial resolution of the system is ∼450 nm (pump wavelength = 792 nm). The spectra were corrected for wavenumber position (*x*-axis calibration) based on a lambda scan of polystyrene–PMMA beads in the region 3060 cm^–1^ (ν(=CH)).

### SRS Imaging and Spectral Phasor Analysis

HepG2 cells were plated on high-precision glass coverslips (#1.5H thickness, 22 × 22 mm, Thorlabs) in a 6-well plate in DMEM at a concentration of 5 × 10^5^ cells per mL and incubated at 37 °C and 5% CO_2_ for a 24 h prior to treatment. Cells were treated with the relevant drug from a stock solution in DMSO (or DMSO as a control) and incubated at 37 °C and 5% CO_2_ for the indicated time. Prior to imaging, the plates were aspirated and washed with PBS (2 × 2 mL), the cells were fixed with paraformaldehyde (4% in PBS, 15 min at rt), and washed with PBS (2 × 2 mL). The coverslips were then affixed to glass microscope slides with a PBS boundary between the glass layers prior to imaging. For live cell imaging, the cells were washed with PBS (2 × 2 mL) following the relevant treatment before mounting onto glass microscope sides as described. Z-stacks were acquired at 1 μm increments in the *Z* plane. SRS imaging was performed in triplicate from three biological replicates for each treatment condition.

### Hyperspectral SRS Imaging

Hyperspectral SRS images were acquired across the range 2800–3050 cm^–1^ using a 0.4 nm retune in the pump beam and 9.75 μs pixel dwell time across a 512 × 512 frame.

### Spectral Phasor Analysis

The SRS image data set across the range 2800–3050 cm^–1^ was imported into ImageJ, and an average intensity projection was created. The spectral phasor analysis was performed as described by Fu et al.^18^ using a plug-in for ImageJ.^21^ The background areas were removed from the image using an threshold intensity mask. Segmentation of the phasor plot was performed manually using regions-of-interest to create images of discrete cellular locations using the spectral phasor analysis of the propranolol and cyclosporin A-treated cells as models for phospholipidosis and steatosis, respectively. The corresponding average spectra for each ROI were plotted using Origin.

## Data Availability

The raw data supporting this research publication will be made available from the University of Strathclyde at the following link: 10.15129/07e39a41-5524-44fe-ac6a-4faf8e35f879

## Supporting Information Available

Experimental procedures for all experiments presented in this article; additional experimental methods; data pertaining to the imaging and analysis of **2**–**4**; experimental synthetic procedures; and ^1^H, ^19^F, and ^13^C NMR spectra and other analytical data for all reported compounds (PDF)
