# Supplementary material for: Label-Free Screening of Drug-Induced Liver Injury Using Stimulated Raman Scattering Microscopy and Spectral Phasor Analysis
Source: Anal Chem. 2024 Jun 18;96(26):10639–47. doi: 10.1021/acs.analchem.4c01285 (PMC11223099; doi:10.1021/acs.analchem.4c01285)

Electronic Supplementary Information

Label-free Screening of Drug-induced Liver Injury Using Stimulated Raman Scattering Microscopy and Spectral Phasor Analysis

William J. Tipping,[a] Liam T. Wilson,‡[b] Nicholas C. O. Tomkinson*[b] Karen Faulds,*[a] and Duncan Graham*[a]

a) Centre for Molecular Nanometrology, WestCHEM, Department of Pure and Applied Chemistry, Technology and Innovation Centre, University of Strathclyde, 99 George Street, Glasgow, G1 1RD, U.K.
e-mail: karen.faulds@strath.ac.uk, duncan.graham@strath.ac.uk

b) Department of Pure and Applied Chemistry, Thomas Graham Building, University of Strathclyde, 295 Cathedral Street, Glasgow, G1 1XL, U.K.
e-mail: nicholas.tomkinson@strath.ac.uk

‡ Present address: The School of Chemistry, Joseph Black Building, The University of Glasgow, Glasgow, G12 8QQ, UK.

Contents

Material and Methods……………………………………………………..……………………2

Figures S1-S7……………………………………..…………………..………………………5

References………………….……………………………………………………………………12

Synthesis and characterisation of propranolol analogues…………...……………………...….13

Materials and Methods

Reagents and chemicals

(±)-Propranolol hydrochloride was purchased from Sigma-Aldrich used as supplied. The alkyne-labelled analogues (**2-4**) were synthesised in house (see page 13). Stock solutions of each compound were prepared at a concentration of 100 mM in anhydrous DMSO. Amiodarone, chlorpromazine, cyclosporin A and tamoxifen were all purchased from Sigma-Aldrich and used as supplied. Stock solutions were prepared at 50 mM in anhydrous DMSO.

Cell culture

HepG2 cells were purchased as an authenticated stock from the European Collection of Authenticated Cell Cultures (ECACC) operated by Public Health England (catalogue number 85011430). HepG2 cells were cultured in Dulbecco's modified Eagle medium low glucose (DMEM containing 1 g/L glucose, GIBCO™, Fisher Scientific) supplemented with 10% foetal bovine serum (FBS, Gibco™, Fisher Scientific), 1% penicillin/streptomycin (Gibco™, 10 000 U mL^{-1}, Fisher Scientific) and 1% amphotericin B (Gibco™, 250 µg mL^{-1}, Fisher Scientific). Cells were maintained at 37 °C and 5% CO_2 in a humidified incubator and were routinely sub-cultured at *ca.* 80% confluency.

SRS microscopy

An integrated laser system (picoEmerald™ S, Applied Physics & Electronics, Inc.) was used to produce two synchronised laser beams at 80 MHz repetition rate. A fundamental Stokes beam (1031.4 nm, 2 ps pulse width) was intensity modulated by an electro-optic-modulator (EoM) with >90% modulation depth, and a tunable pump beam (700–960 nm, 2 ps pulse width, <1 nm (<10 cm^{-1}) spectral bandwidth) was produced by a built-in optical parametric oscillator. The pump and Stokes beams were spatially and temporally overlapped using two dichroic mirrors and a delay stage inside the laser system and coupled into an inverted laser-scanning microscope (Leica TCS SP8, Leica Microsystems) with optimised near-IR throughput. SRS images were acquired using 40× objective (HC PL IRAPO 40×, N.A. 1.10 water immersion lens) with a 9.75–48 µs pixel dwell time over a 512 × 512 or a 1024 × 1024 frame. The Stokes beam was modulated with a 20 MHz EoM. Forward scattered light was collected by a S1 N. A. 1.4 condenser lens (Leica Microsystems). Images were acquired at 12-bit image depth. The laser powers measured after the objective lens were in the range 10–30 mW for the pump beam only, 10–50 mW for the Stokes beam only and 20–70 mW (pump and Stokes beams). The spatial resolution of the system is ~450 nm (pump wavelength = 792 nm). The spectra were corrected for wavenumber position (*x*-axis calibration) based on a lambda scan of polystyrene–PMMA beads in the region 3060 cm^{-1} (v(=CH)).

SRS imaging and spectral phasor analysis

HepG2 cells were plated on high precision glass coverslips (#1.5H thickness, 22 × 22 mm, Thorlabs) in a 6-well plate in DMEM at a concentration of 5 × 10^5 cells per mL and incubated at 37 °C and 5% CO_2 for a 24 h prior to treatment. Cells were treated with the relevant drug from a stock solution in DMSO (or DMSO as a control) and incubated at 37 °C and 5% CO_2 for the indicated time. Prior to imaging, the plates were aspirated and washed with PBS (2 × 2 mL), the cells were fixed with paraformaldehyde (4% in PBS, 15 min at rt), and washed with PBS (2 × 2 mL). The coverslips were then affixed to glass microscope slides with a PBS boundary between the glass layers prior to imaging following the method described in Ref. 1. For live cell imaging, the cells were washed with PBS (2 × 2 mL) following the relevant treatment, before mounting onto glass microscope sides as described. Z-stacks were acquired at 1 µm increments in the *Z* plane.

Hyperspectral SRS imaging

Hyperspectral SRS images were acquired across the range 2800-3050 cm^{-1} using a 0.4 nm re-tune in the pump beam and 9.75 μs pixel dwell time across a 512×512 frame.

Raman spectroscopy

All Raman spectra were acquired on a Renishaw inVia Raman microscope equipped with a 532 nm Nd:YAG laser providing a maximum output at source of 50 mW and using a 1800 lines per mm grating. Prior to spectral acquisition, the instrument was calibrated using the internal silicon standard at 520.5 cm^{-1}. Live-cell Raman imaging: HepG2 cells were plated on glass bottomed culture dishes (35 mm high, Ibidi) at a concentration of 2.5 × 10^5 cells per mL and incubated at 37 °C and 5% CO_2 for 24 h prior to treatment. Cells were treated with propranolol or propranolol analogue (**2-4**) from 100 mM stock solution in DMSO (or DMSO as a control) and incubated at 37 °C and 5% CO_2 for the indicated time. Prior to imaging, the dishes were aspirated and washed with PBS (2 × 2 mL) and imaged in PBS. Raman maps were acquired using λ = 532 nm with a Nikon 60×, N.A. 1.0 NIR Apo water immersion objective, 1 μm step size in x and y, 0.5 s acquisition time, 50% laser power (*ca.* 18 mW) and a spectral centre of 2800 cm^{-1} (high wavenumber region) or 1500 cm^{-1} (fingerprint region). Three replicate maps of different cells were acquired from a single culture plate for each condition. Ratiometric images were prepared using a custom script on MATLAB as reported previously (Ref. 2).

LipidTOX imaging using fluorescence microscopy

For imaging DIPL and DIS using fluorescence microscopy, the HCS LipidTOX™ Phospholipidosis and Steatosis Detection Kit, for high-content screening, for cellular imaging was used. Briefly, HepG2 cells were plated on high precision glass coverslips (#1.5H thickness, 22 × 22 mm, Thorlabs) in a 6-well plate in DMEM at a concentration of 5 × 10^5 cells per mL and incubated at 37 °C and 5% CO_2 for a 24 h prior to treatment. Cells were treated with propranolol or propranolol analogue (**2-4**) from a 100 mM stock solution in DMSO (or DMSO as a control) and incubated at 37 °C and 5% CO_2 for the indicated time. For imaging DIPL, cells were treated with LipidTOX Red for 48 h with the indicated drug as per the manufacturer's guideline (ThermoFisher Scientific). The cells were fixed with paraformaldehyde (4% in PBS, 15 min at rt), and washed with PBS (2 × 2 mL). For imaging DIS, the cells were then treated with LipidTOX Green for 30 mins, before counterstaining with DAPI (5 mins). Prior to imaging, the plates were aspirated and washed with PBS (2 × 2 mL), The coverslips were then affixed to glass microscope slides with a PBS boundary between the glass layers prior to imaging following the method described in ref. 1. Fluorescence imaging was achieved using a 63× lens with excitation at 405 nm (DAPI), 488 nm (LipidTOX Green) and 561 nm (LipidTOX Red). Mean fluorescence intensity per cell was determined using ImageJ.

Data processing

SRS images

False colour assignments, scale bars and image overlays were added to images using ImageJ software. Consistent brightness and contrast settings were used when comparing image datasets. For the lipid droplet analysis, a Z-stack of SRS images at 2851 cm^{-1} (CH_2, lipids) was acquired across a typical field of view for each treatment condition and a maximum intensity projection generated using ImageJ. The mean SRS intensity per cell was determined by first, manually selecting individual cells using the freehand selection tool, and the intensity determined using ImageJ analysis tool. For CH_2/CH_3 ratio imaging, a threshold (mask) image was first generated by adjusting threshold on ImageJ, then non-zero values were normalized

to one. CH_2 images were then divided by the corresponding CH_3 image, and the resulting ratio image multiplied with the mask image to create the final CH_2/CH_3 ratio image. The display range of CH_2/CH_3 ratio images is set to be 0–1 and are presented in the Rainbow RGB LUT.

Spectral phasor analysis

The SRS image data set across the range 2800–3050 cm^{-1} was imported into ImageJ and an average intensity projection was created. The spectral phasor analysis was performed as described by Fu *et al.* (Ref. 3) using a plug-in for ImageJ (see Ref. 4). The background areas were removed from the image using an threshold intensity mask as described above. Segmentation of the phasor plot was performed manually using regions-of-interest to create images of discrete cellular locations. The corresponding average spectra for each ROI are plotted using Origin.

Raman spectra

All spectra were processed using WiRE 4.4™. Spectral baselines were subtracted using an 11th order polynomial fitting on WiRE 4.4™. The spectra were normalised using Origin2018 software. Peak centres and intensity values were ascertained using the Lorentzian function built into the curve-fit tool. For cell maps, cosmic rays were removed using a nearest neighbour algorithm and noise filtering was carried out prior to other processing. Average spectra were calculated for each cell map in WiRE 4.4™, from which spectra and output images were extracted.

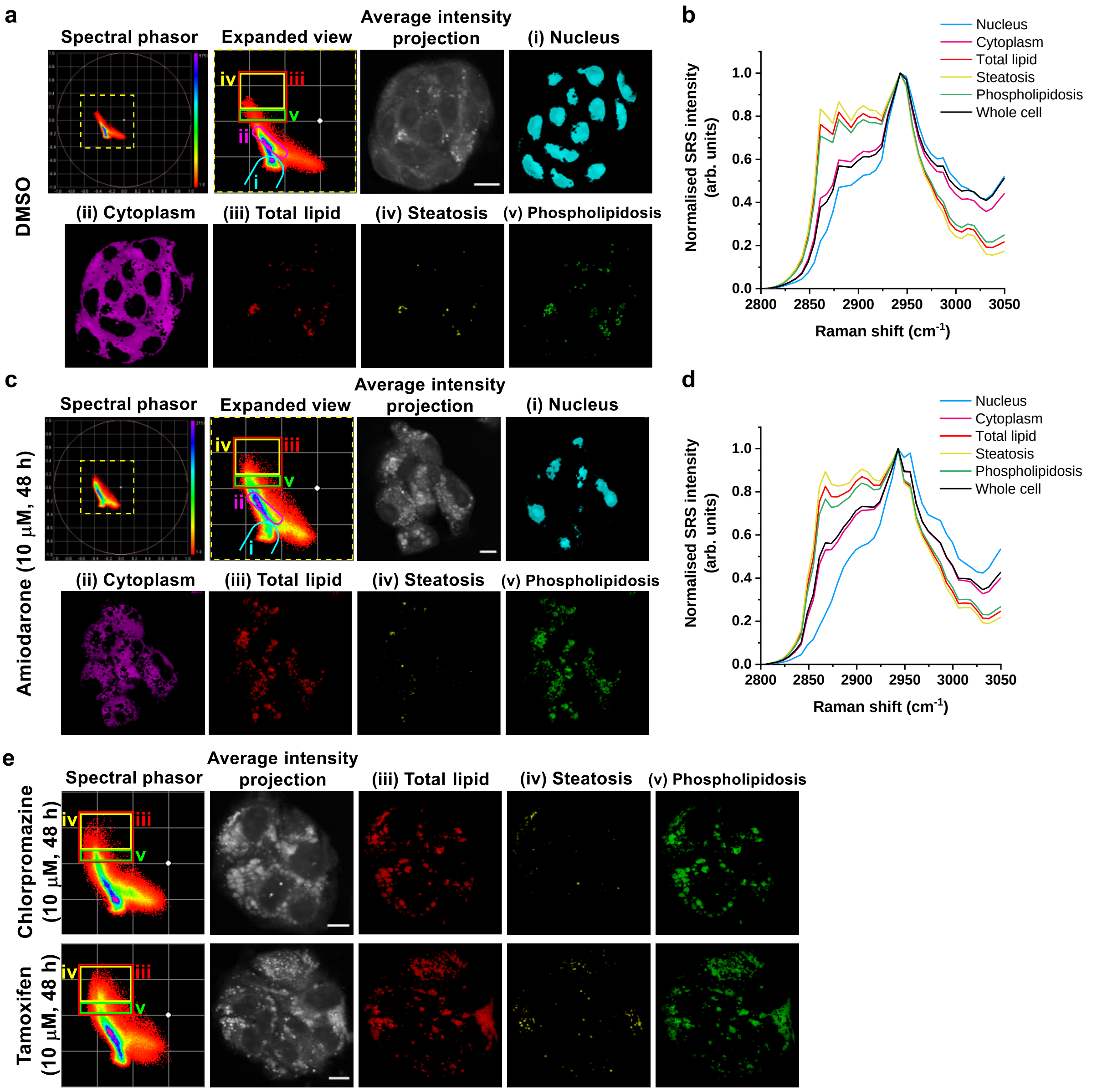

Figure S1 Hyperspectral SRS imaging and spectral phasor analysis of drug-induced liver injury in clinical drug candidates. HepG2 cells were treated with DMSO (**a**, control) or selected drug treatment (**c** amiodarone; **e** chlorpromazine or tamoxifen) at the indicated concentrations before hyperspectral SRS images were acquired across the range 2800-3050 cm^{-1} (0.4 nm re-tune, 40 images). A spectral phasor analysis of the hsSRS image stack is provided. The yellow dashed marker represents the area selected in the expanded view, which has been segmented based on the colour-coded markers into (i) nucleus, (ii) cytoplasm, (iii) total lipid, (iv) steatosis, (v) phospholipidosis. An average intensity projection is also provided (scale bar: 10 μm). For the DMSO control, **a**, and amiodarone treated cells, **c**, average SRS spectra corresponding to the ROIs (i)-(v) are provided in **b** and **d**, respectively.

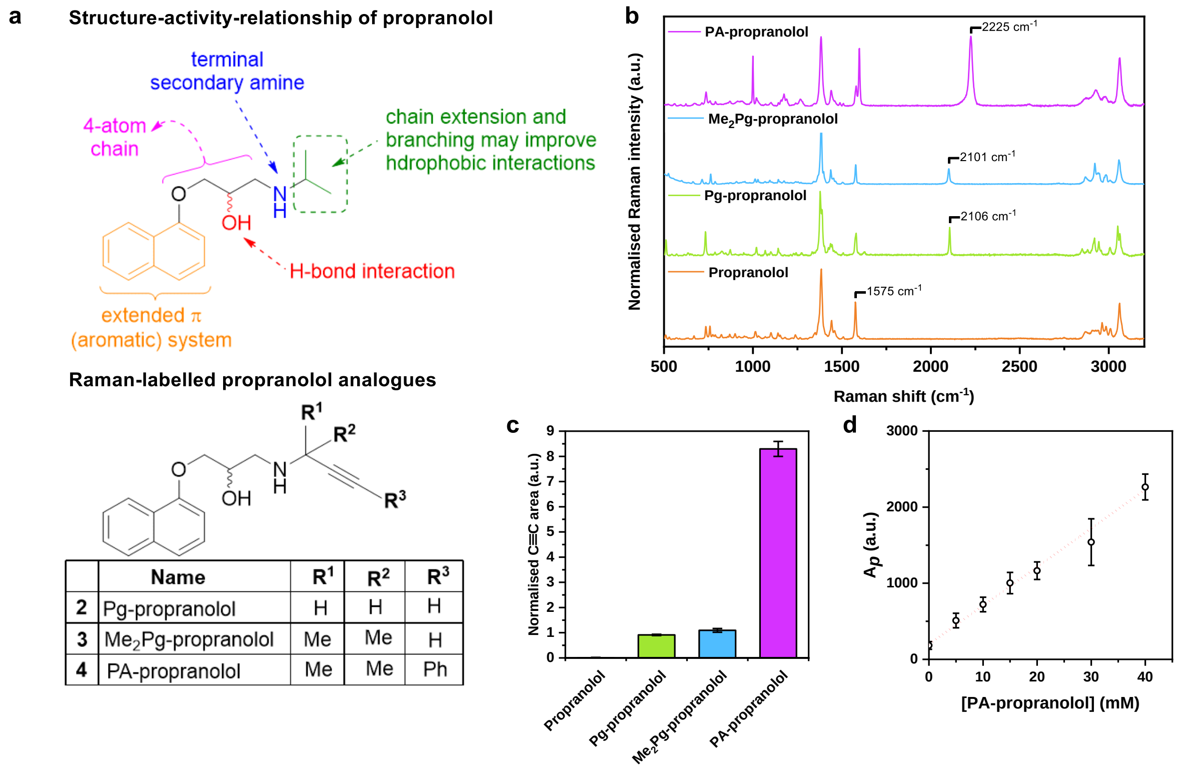

	Name	R^1	R^2	R^3
2	Pg-propranolol	H	H	H
3	Me_2Pg-propranolol	Me	Me	H
4	PA-propranolol	Me	Me	Ph

Figure S2 Development of alkyne labelled propranolol analogues. **a** Structure-activity-relationship assessment of propranolol **1**. Tabulated chemical structures of propranolol analogues (**2-4**) described in this study. **b** Raman spectral analysis of propranolol and the alkyne labelled analogues. Raman spectra were acquired for each compound in solid form and normalised to the intensity of the peak at 1575 cm^{-1} (C-H bending naphthalene ring). Raman spectra were acquired using a 532 nm laser, 10s acquisition time, 20× objective lens (~3.6 mW). **c** Integration analysis of the alkyne peaks within the cell-silent region (1800 – 2800 cm^{-1}) of the Raman spectra in **a**. Data represent mean peak area with error bars ±S.D. of three replicate spectra. **d** Calibration curve of PA-propranolol in DMSO. Raman spectra were acquired using 532 nm excitation, 0.5 s acquisition time, 50× objective lens (~17 mW). Data represent the mean peak area at 2225 cm^{-1} (A_p) with error bars ±S.D.

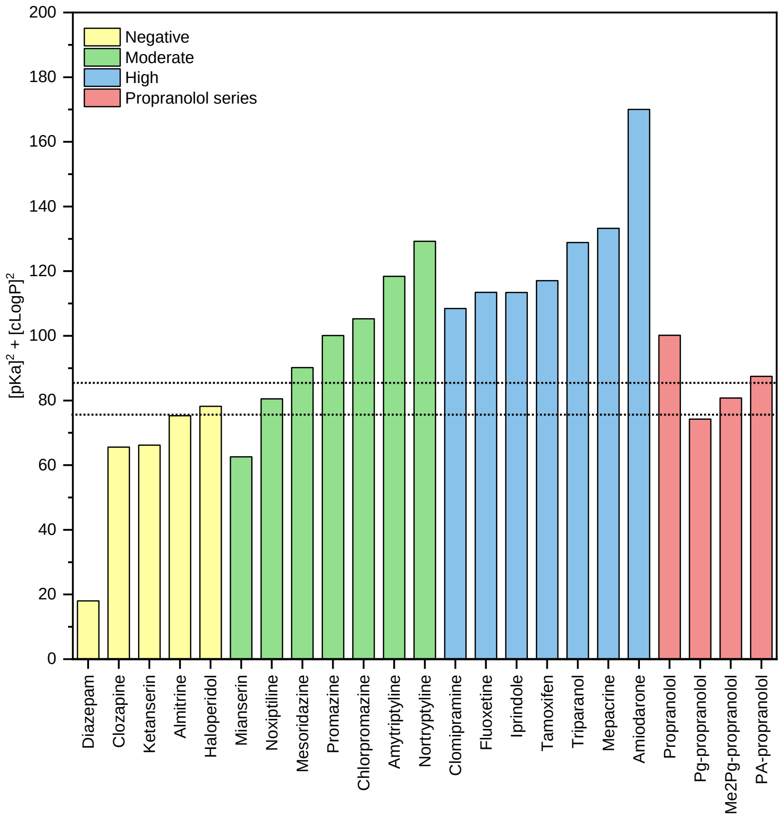

Figure S3 Analysis of the physiochemical properties, cLogP and pKa, of drug molecules with phospholipidosis inducing capacity. The calculated LogP and pKa were predicted using MarvinSketch and the properties of the propranolol series (propranolol, pg-propranolol, Me_2-propranolol and PA-propranolol) were compared to drugs categorised as negative, moderate- (detected in cell cultures) and high-DIPL (detected in cell culture and animal models) as described in Ref. 5. Dashed lines indicate the region at which the model is unreliable for predicting DIPL.

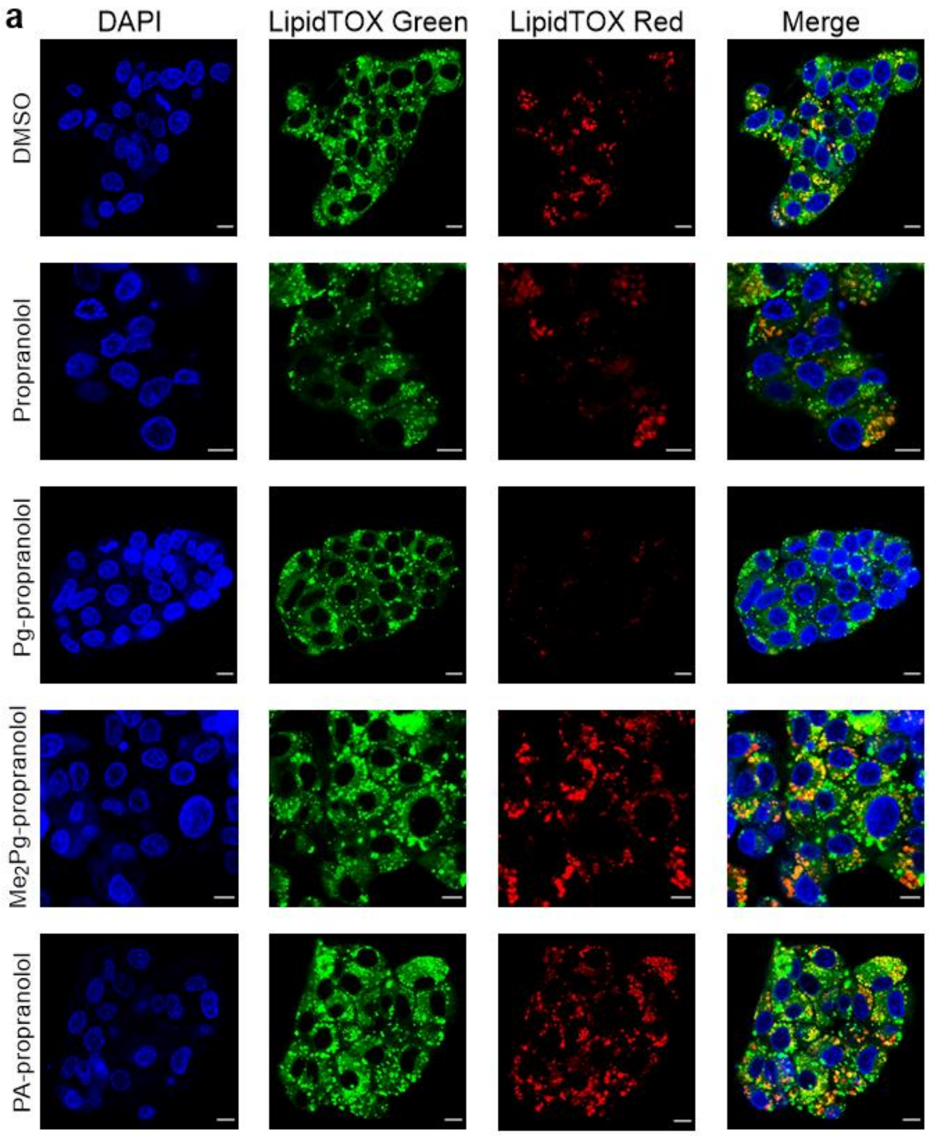

Figure S4 Investigating DIS and DIPL following treatment with propranolol or an alkyne labelled analogue. HepG2 cells were treated with propranolol (30 µM, 48h), an alkyne labelled analogue (30 µM, 48h) or DMSO control. In each sample, a concomitant treatment with LipidTOX Red (48 h) was performed before fixing (4% PFA, 15 min, 37 °C) and staining with LipidTOX Green (30 min) and DAPI according to manufacturer's recommendation. Scale bars: 10 µm.

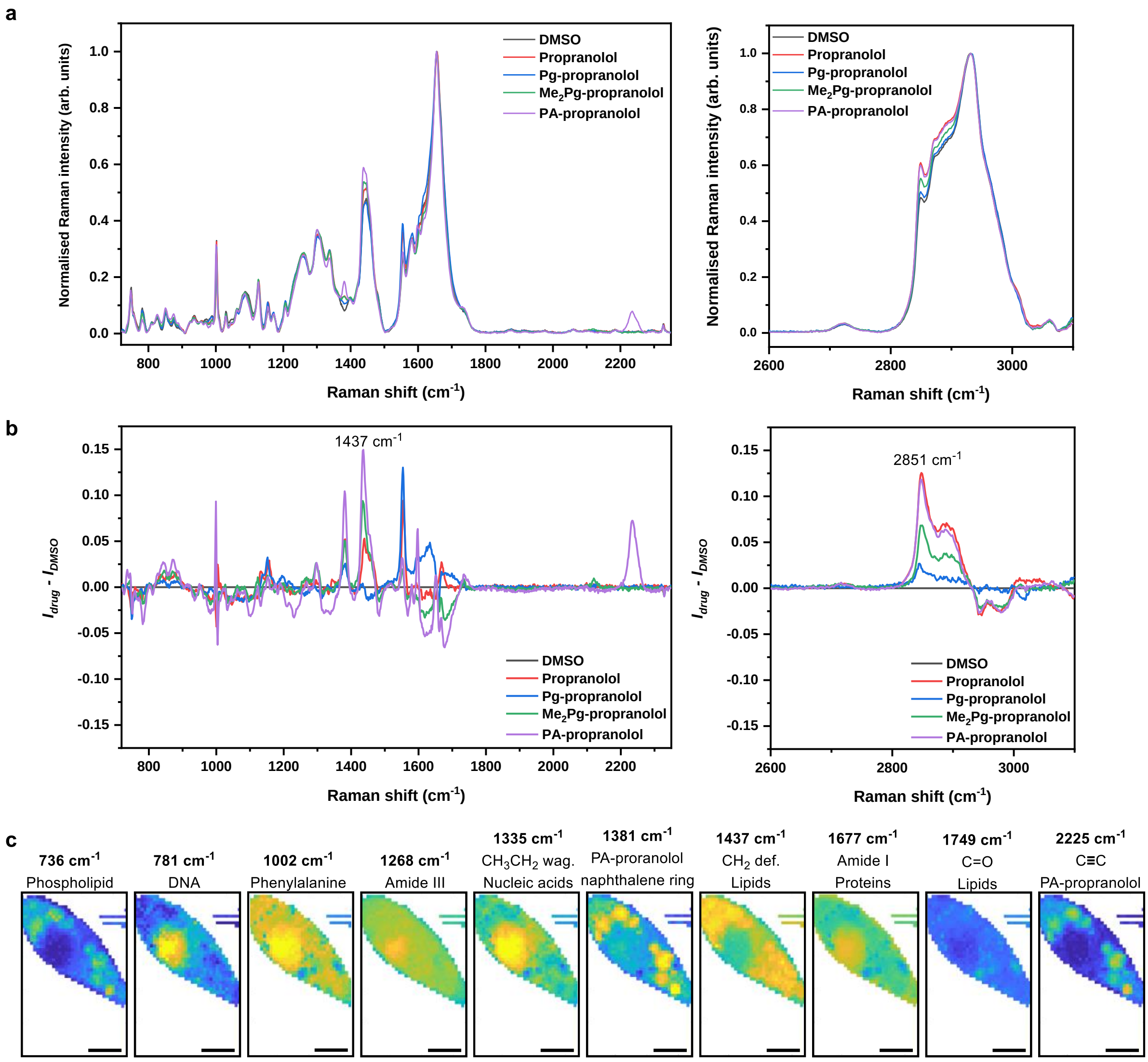

Figure S5 Ratiometric Raman imaging of DIPL. **a** Raman spectral analysis of HepG2 cells treated with DMSO (control), propranolol (30 μM, 48h) or an alkyne-labelled propranolol analogue (30 μM, 48h). The mean Raman spectra extracted from triplicate biological repeats of cell regions of Raman maps are presented for the fingerprint region (750-2340 cm^{-1}) and the high-wavenumber region (2600-3100 cm^{-1}). The spectra were acquired using 532 nm laser excitation for 0.5s using a 60× objective lens (~17 mW) and a 1 μm pixel size. **b** Difference spectra for the Raman spectra presented in **a**. The Raman intensity of the DMSO treated cells is subtracted from the drug-treated cells to highlight differences in the overall spectral intensities for each Raman peak in the fingerprint and high-wavenumber regions. **c** Ratiometric Raman images of a live HepG2 cell treated with PA-propranolol (30 μM, 48h). The Raman maps were acquired as per **a**, and the ratio maps generated from the peak of interest normalised to the intensity of the 1655 cm^{-1} (amide-I) intensity. Scale bars: 10 μm

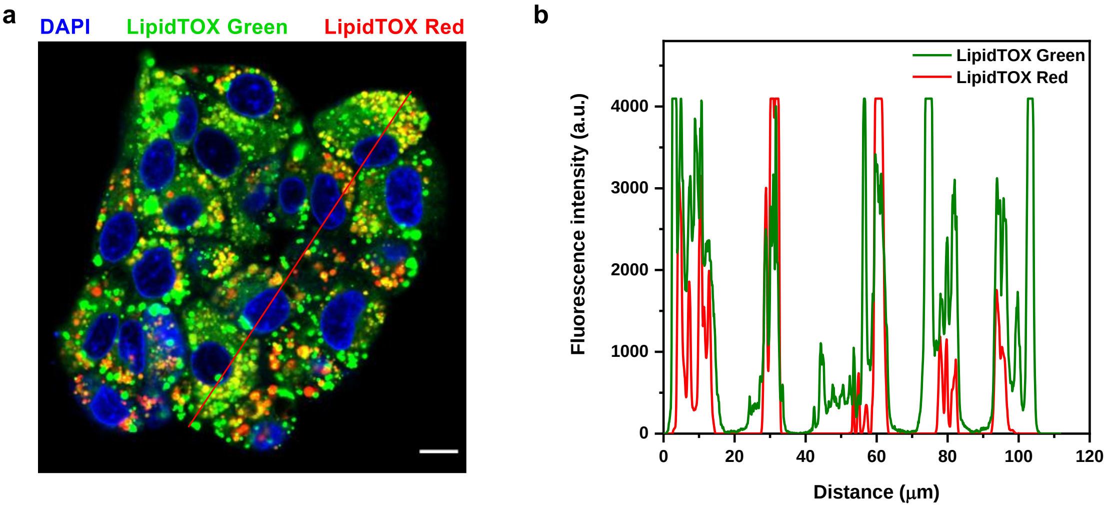

Figure S6 Colocalization analysis of LipidTOX Green and LipidTOX Red signals in HpG2 cells. HepG2 cells were treated with PA-propranolol (30 μM, 48h) and LipidTOX Red (48 h) before fixing and staining with LipidTOX Green and counterstaining with DAPI and imaging using a confocal fluorescence microscope. **a** Merged image of the three fluorescent stains. **b** line plot analysis of signal intensity for the LipidTOX Green and LipidTOX Red signal across the red line marked in **a** highlighting areas of co-localisation between the two stains.

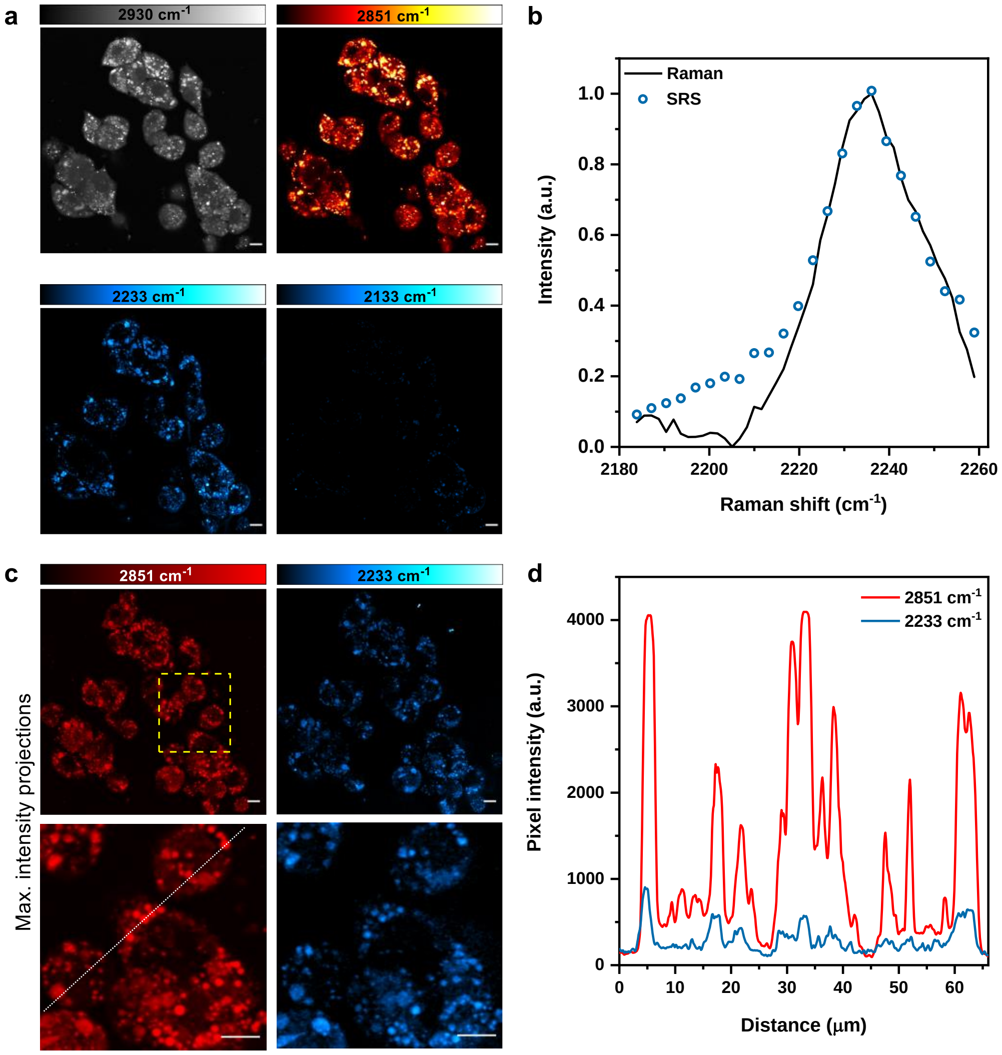

Figure S7 Live cell visualisation of PA-propranolol distribution using SRS microscopy. **a** HepG2 cells were treated with PA-propranolol (30 µm, 48h) before live cell imaging using SRS microscopy at the following frequencies: 2930 cm^{-1} (CH_3 symmetric stretch, greyscale LUT), 2851 cm^{-1} (CH_2 symmetric stretch, red hot LUT), 2233 cm^{-1} (C≡C, PA-propranolol, cyan hot LUT) and 2133 cm^{-1} (cell-silent region, cyan hot). Scale bars: 10 µm. **b** The Raman spectrum of PA-propranolol and SRS spectrum of PA-propranolol from HepG2 cells treated with PA-propranolol (30 µm, 48h). The Raman spectrum was acquired using a 532 nm laser for 0.5 s using a 60× objective lens (~17 mW). SRS spectrum were acquired by re-tuning the laser wavelength in 0.4 nm increments between image frames. **c** Maximum intensity projections of the population of cells presented in **a**. The z focal plane was adjusted by 1 µm in between image frames. The stacks were recorded across the same volume at 2851 cm^{-1} and 2233 cm^{-1}. A yellow dashed marker indicates the area presented in the expanded view (bottom image pairs) and a white line indicates the intensity plot profile presented in **d**.

References

1) D. Fu, J. Zhou, W. S. Zhu, P. W. Manley, Y. K. Wang, T. Hood, A. Wylie and X. S. Xie, *Nat. Chem.*, **2014**, *6*, 614-622.
2) L. E. Jamieson, C. Wetherill, K. Faulds and D. Graham, *Chem. Sci*., **2018**, *9*, 6935-6943.
3) D. Fu and X. S. Xie, *Anal. Chem*., **2014**, *86*, 4115-4119.
4) W. J. Tipping, L. T. Wilson, C. An., A. A. Leventi, A. W. Wark, C. Wetherill, N. C. O. Tomkinson, K. Faulds and D. Graham, *Chem. Sci*., **2022**, *13*, 3468-3476.
5) L. Zhou, G. Geraci, S. Hess, L. Yang, J. Wang, and U. Argikar, *Anal. Chem*. **2011**, *83*, 6980-6987

Synthesis

All reagents were obtained from commercial sources, including Sigma-Aldrich, Alfa Aesar and Fluorochem and used without purification unless otherwise stated. The anhydrous solvents tetrahydrofuran (THF), dichloromethane (CH_2Cl_2), diethyl ether (Et_2O), hexane and toluene were obtained from a PureSolv MD 5 Solvent Purification System by Innovative Technology Inc., and handled under inert atmosphere without further purification. Other solvents were acquired from commercial sources and used without further purification unless otherwise stated. Flash chromatography was carried out using Fischer Scientific chromatography grade silica 60 Å particle size 35–70 micron. Analytical thin layer chromatography was carried out using aluminium-backed plates coated with Machery-Nagel pre-coated TLC sheets, coated in 0.20 mm silica gel 60 with UV254 fluorescent indicator. Sheets were visualized under UV light (at 254 nm) or stained using p-anisaldehyde. Nuclear magnetic resonance (NMR) spectra were recorded on a Bruker Avance III spectrometer operating at 400 MHz (^{1}H) and 101 MHz (^{13}C), or Bruker Avance 500 spectrometer, operating at 500 MHz (^{1}H) and 125 MHz (^{13}C). Chemical shifts were reported in parts per million (ppm) in the scale relative to $CDCl_3$, 7.26 ppm for ^{1}H NMR and 77.16 for ^{13}C NMR; $(CD_3)_2SO$ (dimethylsulfoxide), 2.50 ppm for ^{1}H NMR and 39.52 for ^{13}C NMR; $(CD_3)_2CO$, 2.05 for ^{1}H NMR and 29.84 for ^{13}C NMR. Multiplicities are abbreviated as: s, singlet; d, doublet; t, triplet; q, quartet; dd, doublet of doublets; ddd, doublet of doublets of doublets; td, triplet of doublets; app.t, apparent triplet; app.td, apparent triplet of doublets; app.p, apparent pentet; hept, heptet; dhept, doublet of heptets; m, multiplet; br, broad. Coupling constants are measured in Hertz (Hz). Low-resolution mass spectra (LRMS) were recorded on an Agilent 6130 single quadrupole with APCI/ESI dual source, on a ThermoQuest Finnigan LCQ DUO electrospray, or on an Agilent 7890A GC system equipped with a 30 m DB5MS column connected to a 5975C inert XL CI MSD with TripleAxis Detector and were determined using atmospheric pressure chemical ionization (APCI) unless otherwise stated. ESI refers to electrospray ionization, CI refers to chemical ionization (methane) and EI refers to electron ionization. Melting points were obtained on a Stuart SMP11 device. Infrared spectra were recorded in the range 4000–600 cm^{-1} on a Shimadzu IRAffinity-1 equipped with an ATR accessory. In vacuo refers to evaporation under reduced pressure using a rotary evaporator connected to a diaphragm pump, followed by the removal of trace volatiles using a high vacuum (oil) pump.

General Procedure A

A flame dried microwave vial was charged with epoxide **S1** (1.50 mmol) and the vial was sealed and purged with N_2. Anhydrous EtOH (5 mL) was added, followed by the relevant primary amine (6.00 mmol) and the reaction was heated to 80 °C for 4 h, with stirring. After cooling to ambient temperature, the reaction mixture was evaporated *in vacuo*. Purification by flash chromatography on silica gel using an appropriate eluent afforded the desired β-aminoalcohol products.

Synthesis of 2-((naphthalen-1-yloxy)methyl)oxirane, S1

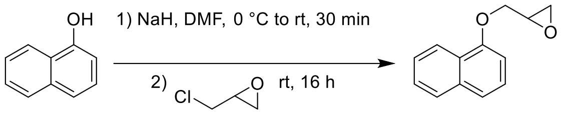

S1

A flame dried round bottomed flask was charged with NaH (60% in mineral oil, 333 mg, 8.32 mmol) and purged with Ar. The contents were suspended in anhydrous *N*,*N*-dimethylformamide (DMF, 15 mL) and cooled 0 °C with stirring. A solution of 1-naphthol (1.00 g, 6.94 mmol) in anhydrous DMF (20 mL) was added dropwise and the reaction was stirred for 30 min at 0 °C before warming to rt. At this point, epichlorohydrin (2.80 mL, 34.7 mmol) was added dropwise and stirring was continued at rt for 16 h. The reaction was quenched with water (200 mL) and extracted with Et_2O (4 x 50 mL). The combined organics were washed with brine (20 mL), dried over Na_2SO_4 and evaporated *in vacuo*. The resulting oil was purified by flash chromatography using sequential solvents (hexane, 50% DCM/petroleum ether 40–60) to afford the desired product **S1** as a colourless oil (1.16 g, 5.79 mmol, 83%). ^{1}H NMR (500 MHz, $CDCl_3$) δ 8.34–8.28 (m, 1H), 7.83–7.77 (m, 1H), 7.53–7.43 (m, 3H), 7.37 (app.t, *J* = 7.9 Hz, 1H), 6.82 (d, *J* = 7.9 Hz, 1H), 4.41 (dd, *J* = 11.0, 3.1 Hz, 1H), 4.17 (dd, *J* = 11.0, 5.5 Hz, 1H), 3.54–3.46 (m, 1H), 2.97 (t, *J* = 4.5 Hz, 1H), 2.86 (dd, *J* = 4.5, 2.6 Hz, 1H); ^{13}C NMR (126 MHz, $CDCl_3$) δ 154.4, 134.7, 127.6, 126.7, 125.9, 125.8, 125.5, 122.2, 121.0, 105.2, 69.1, 50.4, 44.9; FTIR (ATR, cm^{-1}) vmax: 3051, 2995, 2922, 1593, 1578, 1506, 1395; LRMS: (ES + APCI) *m/z* calc. 200.1, found 201.1 $[M+H]^+$.

Synthesis of 1-(naphthalen-1-yloxy)-3-(prop-2-yn-1-ylamino)propan-2-ol, 2

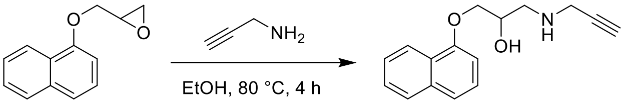

S1 **2**

According to General Procedure A: epoxide **S1** (300 mg, 1.50 mmol) and propargylamine (380 µL, 6.00 mmol) in anhydrous EtOH (5 mL) were stirred at 80 °C for 4 h. Purification by flash chromatography (0–20% (4:1 EtOAc:EtOH)/petroleum ether 40–60) afforded the desired product **2** as a white solid (302 mg, 1.18 mmol, 79%). ^{1}H NMR (400 MHz, $CDCl_3$) δ 8.30–8.21 (m, 1H), 7.85–7.76 (m, 1H), 7.54–7.42 (m, 3H), 7.37 (app.t, *J* = 7.9 Hz, 1H), 6.83 (d, *J* = 7.9 Hz, 1H), 4.32–4.13 (m, 3H), 3.52 (d, *J* = 1.5 Hz, 2H), 3.10 (dd, *J* = 12.0, 2.4 Hz, 1H), 2.97 (dd, *J* = 12.0, 7.1 Hz, 1H), 2.32 (s, 2H), 2.26 (t, *J* = 1.5 Hz, 1H); ^{13}C NMR (101 MHz, $CDCl_3$) δ 154.4, 134.7, 127.7, 126.6, 126.0, 125.7, 125.4, 121.9, 120.9, 105.1, 81.9, 71.9, 70.7, 68.8, 51.1, 38.4; FTIR (ATR, cm^{-1}) vmax: 3294, 3279, 3007, 2916, 2883, 2692, 1730, 1580, 1508, 1458, 1273; LRMS: (ES + APCI) *m/z* calc. 255.1, found 256.1 $[M+H]^+$; HRMS (ESI): $[M+H]^+$ calc. for $C_{16}H_{17}NO_2$ 256.1332, found 256.1338; mp: 80–82 °C.

Synthesis of 1-((2-methylbut-3-yn-2-yl)amino)-3-(naphthalen-1-yloxy)propan-2-ol, 3

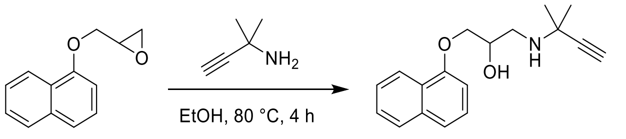

S1 **3**

According to General Procedure A: epoxide **S1** (300 mg, 1.50 mmol) and 2-methylbut-3-yn-2-amine (630 µL, 6.00 mmol) in anhydrous EtOH (5 mL) were stirred at 80 °C for 4 h. Purification by flash chromatography (0–10% (4:1 EtOAc:EtOH)/petroleum ether 40 – 60) afforded the desired product **3** as a white solid (406 mg, 1.43 mmol, 96%). ^{1}H NMR (400 MHz, $CDCl_3$) δ 8.31–8.23 (m, 1H), 7.85–7.77 (m, 1H), 7.54–7.42 (m, 3H), 7.37 (app.t, *J* = 7.9 Hz, 1H), 6.84 (d, *J* = 7.9 Hz, 1H), 4.27–4.16 (m, 3H), 3.15–3.07 (m, 1H), 3.05–2.96 (m, 1H), 2.34 (br.s, 2H), 2.32 (s, 1H), 1.43 (s, 3H), 1.42 (s, 3H); ^{13}C NMR

(101 MHz, $CDCl_3$) δ 154.5, 134.7, 127.7, 126.6, 126.0, 125.7, 125.4, 122.0, 120.8, 105.1, 88.6, 70.9, 70.2, 69.3, 49.7, 46.9, 29.9, 29.6; FTIR (ATR, cm^{-1}) vmax: 3298, 3277, 3051, 2976, 2934, 2849, 2754, 1578, 1508, 1458, 1393, 1267; LRMS: (ES + APCI) *m/z* calc. 283.2, found 284.0 $[M+H]^+$; HRMS (ESI): $[M+H]^+$ calc. for $C_{18}H_{21}NO_2$ 284.1645, found 284.1649; mp: 68–70 °C.

Synthesis of 1-((2-methyl-4-phenylbut-3-yn-2-yl)amino)-3-(naphthalen-1-yloxy)propan-2-ol, 4

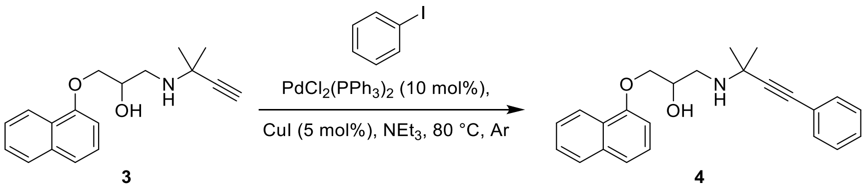

A flame dried microwave vial was charged with alkyne **3** (20 mg, 0.071 mmol), $PdCl_2(PPh_3)_2$ (5 mg, 0.007 mmol) and CuI (0.7 mg, 0.0035 mmol). The vial was then sealed and purged with Ar, before adding anhydrous NEt_3 (0.3 mL) and iodobenzene (12 µL, 0.11 mmol). The mixture was degassed by 3 freeze-pump-thaw cycles, refilling with Ar, before heating to 80 °C for 3 h. After cooling to ambient temperature, the reaction mixture was filtered through celite®, washing with Et_2O (10 mL) and evaporated *in vacuo*. Purification by pipette flash column chromatography (Et_2O) afforded the desired product **4** as a colourless gum (22 mg, 0.061 mmol, 87%). ^1H NMR (500 MHz, $CDCl_3$) δ 8.25 (d, *J* = 8.4 Hz, 1H), 7.80 (d, *J* = 8.4 Hz, 1H), 7.54–7.34 (m, 6H), 7.31–7.25 (m, 3H), 6.85 (d, *J* = 7.6 Hz, 1H), 4.29–4.15 (m, 3H), 3.24–3.17 (m, 1H), 3.13–3.06 (m, 1H), 1.51 (s, 3H), 1.50 (s, 3H); ^{13}C NMR (126 MHz, $CDCl_3$) δ 154.5, 134.7, 131.8, 128.4, 128.0, 127.7, 126.6, 126.0, 125.7, 125.4, 123.4, 122.0, 120.8, 105.1, 94.0, 82.6, 70.9, 69.4, 50.4, 47.1, 30.1, 29.8; FTIR (ATR, cm^{-1}) vmax: 3054, 2924, 2360, 2342, 1596, 1579, 1459, 1442, 1399, 1267, 1240; HRMS (ESI): $[M+H]^+$ calc. for $C_{24}H_{25}NO_2$ 360.1958, found 360.1960.

NMR Data

^{1}H NMR of S1

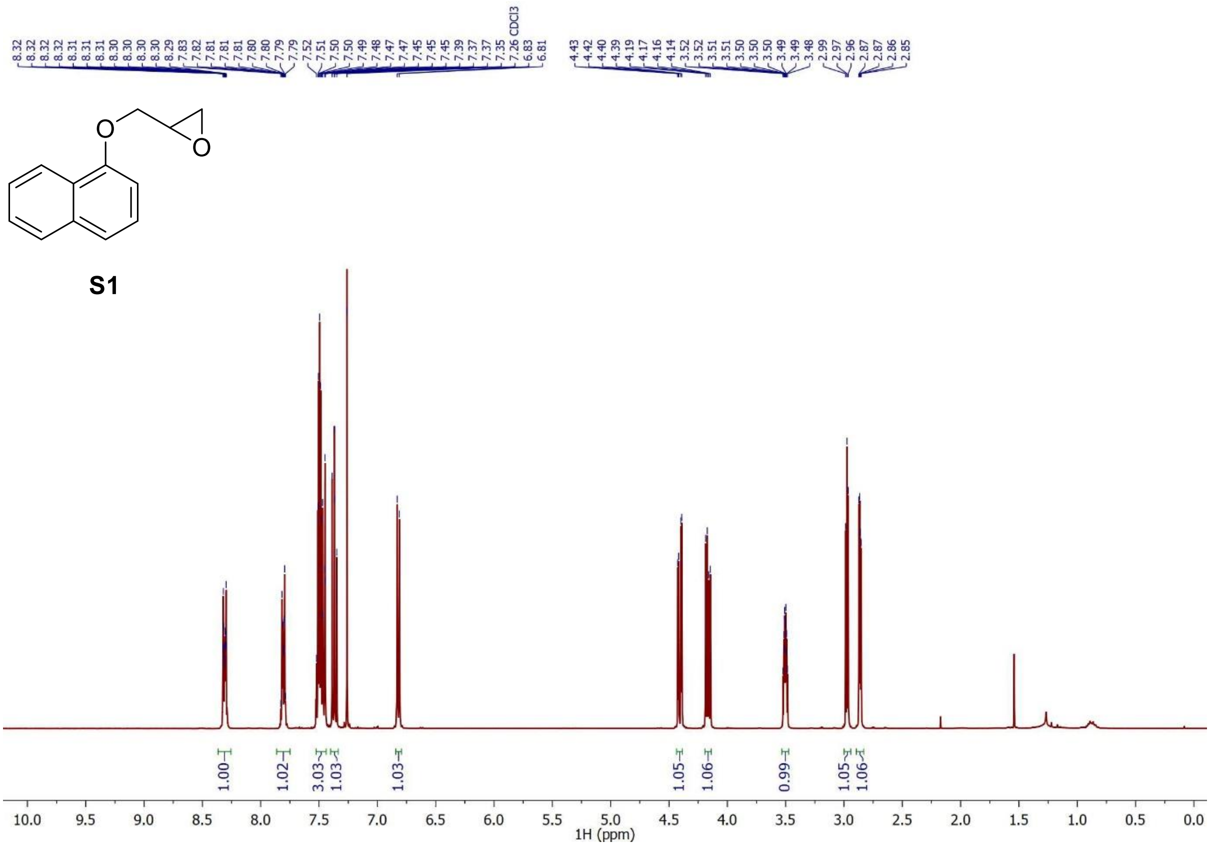

^{13}C NMR of S1

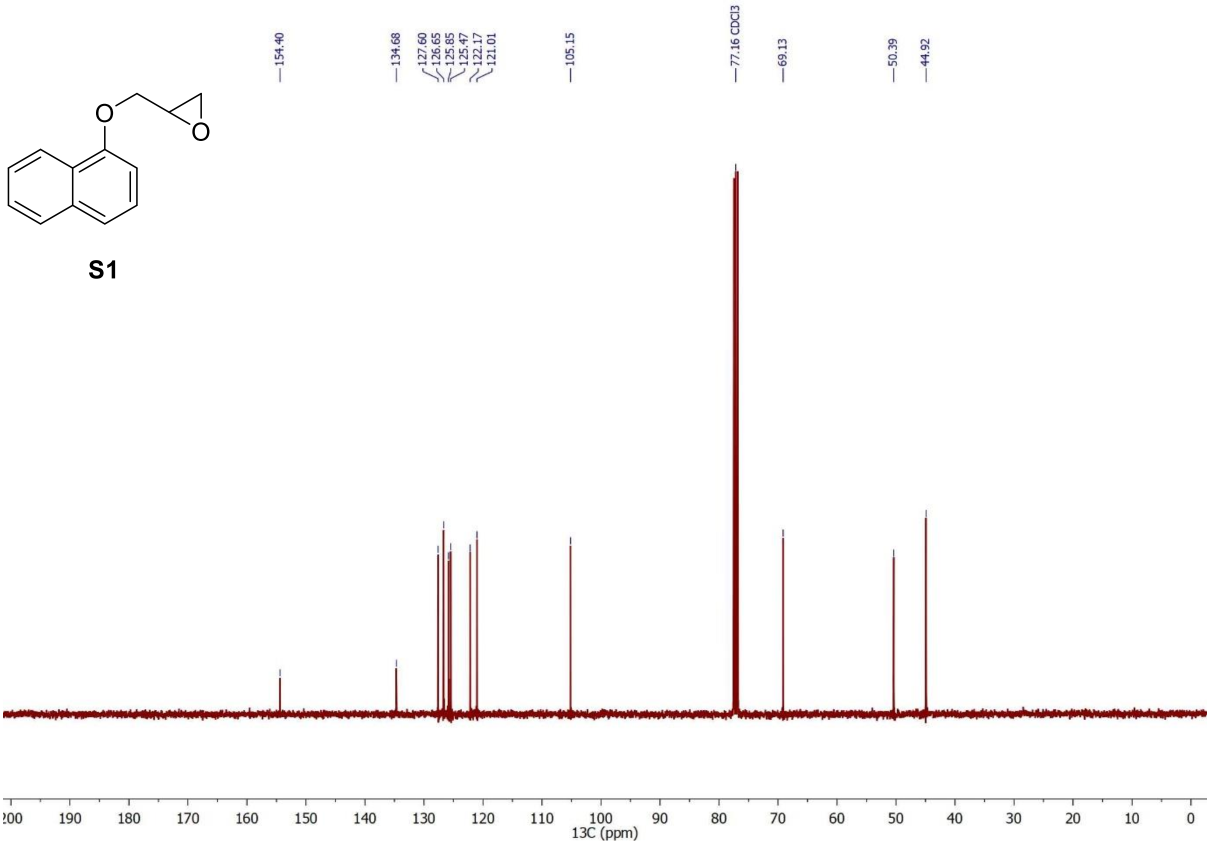

^{1}H NMR of 2

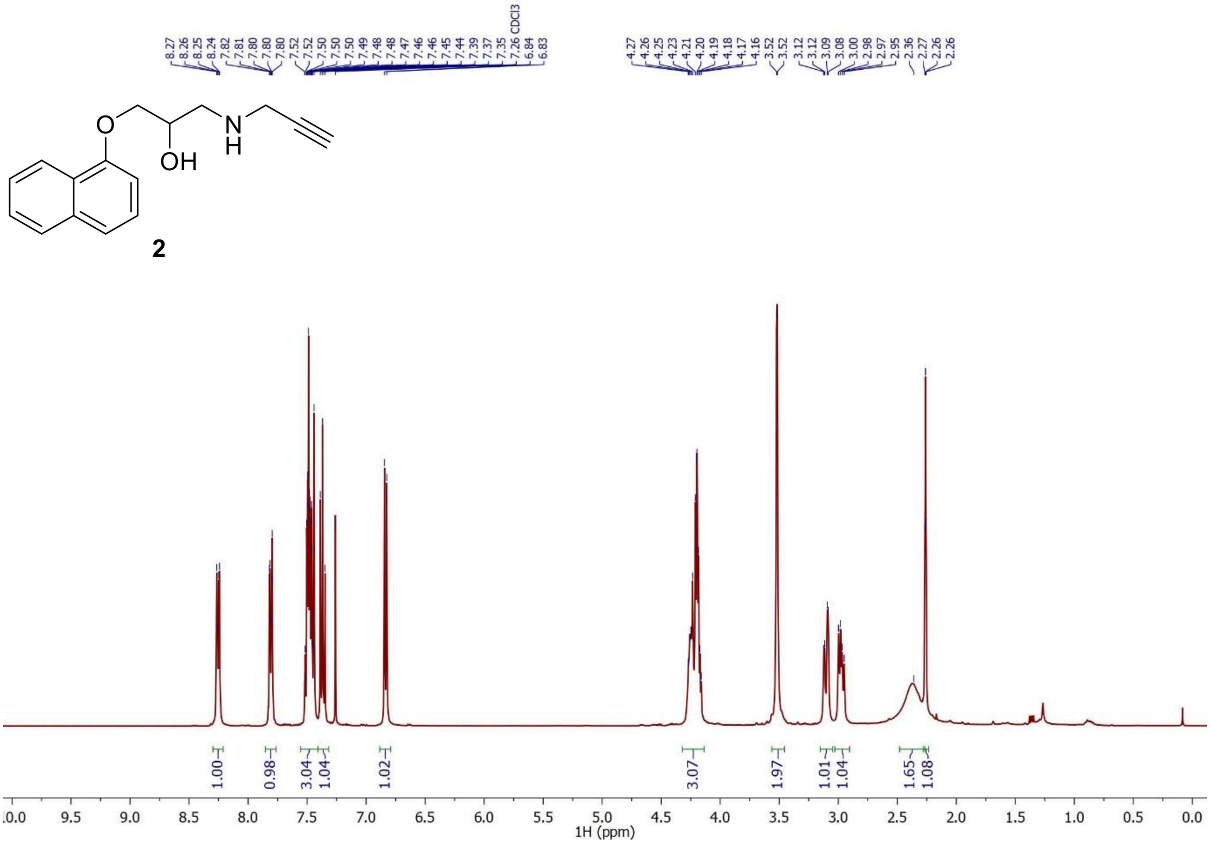

^{13}C NMR of 2

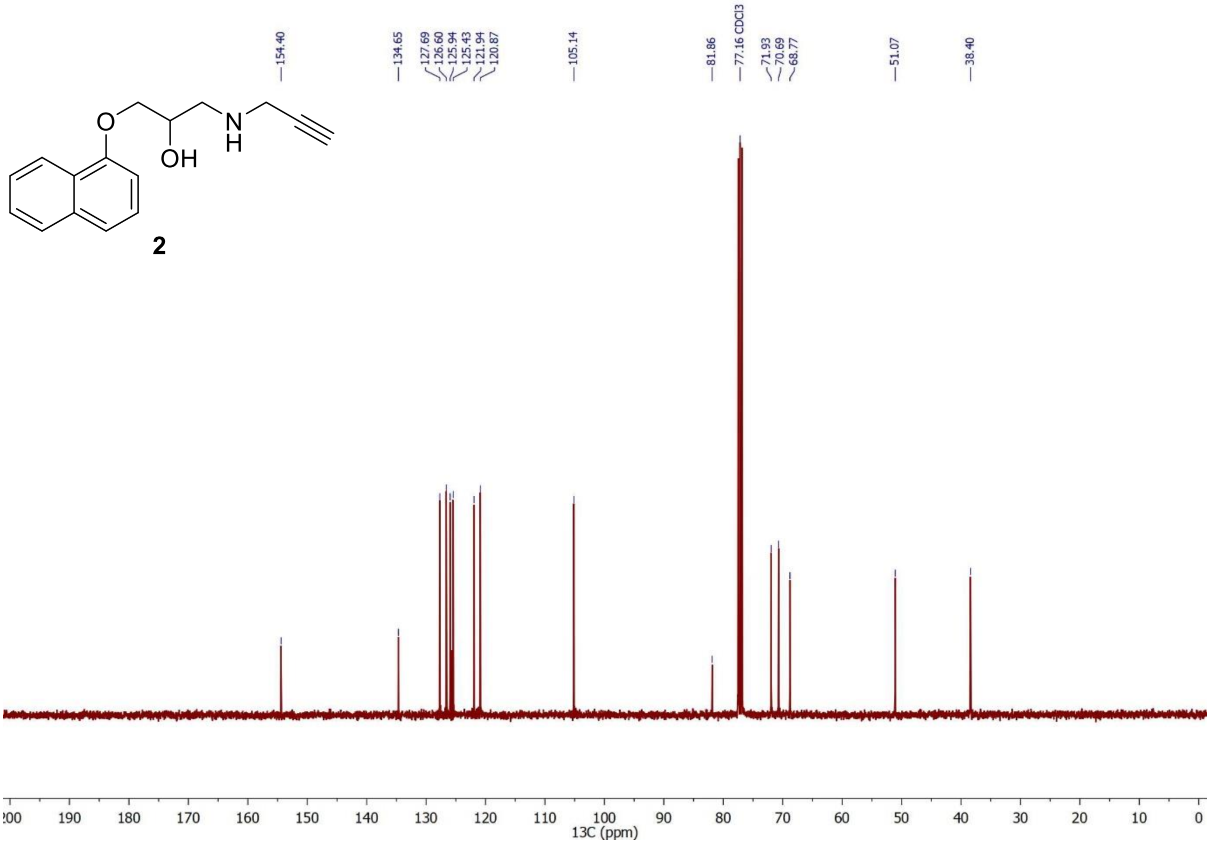

^{1}H NMR of 3

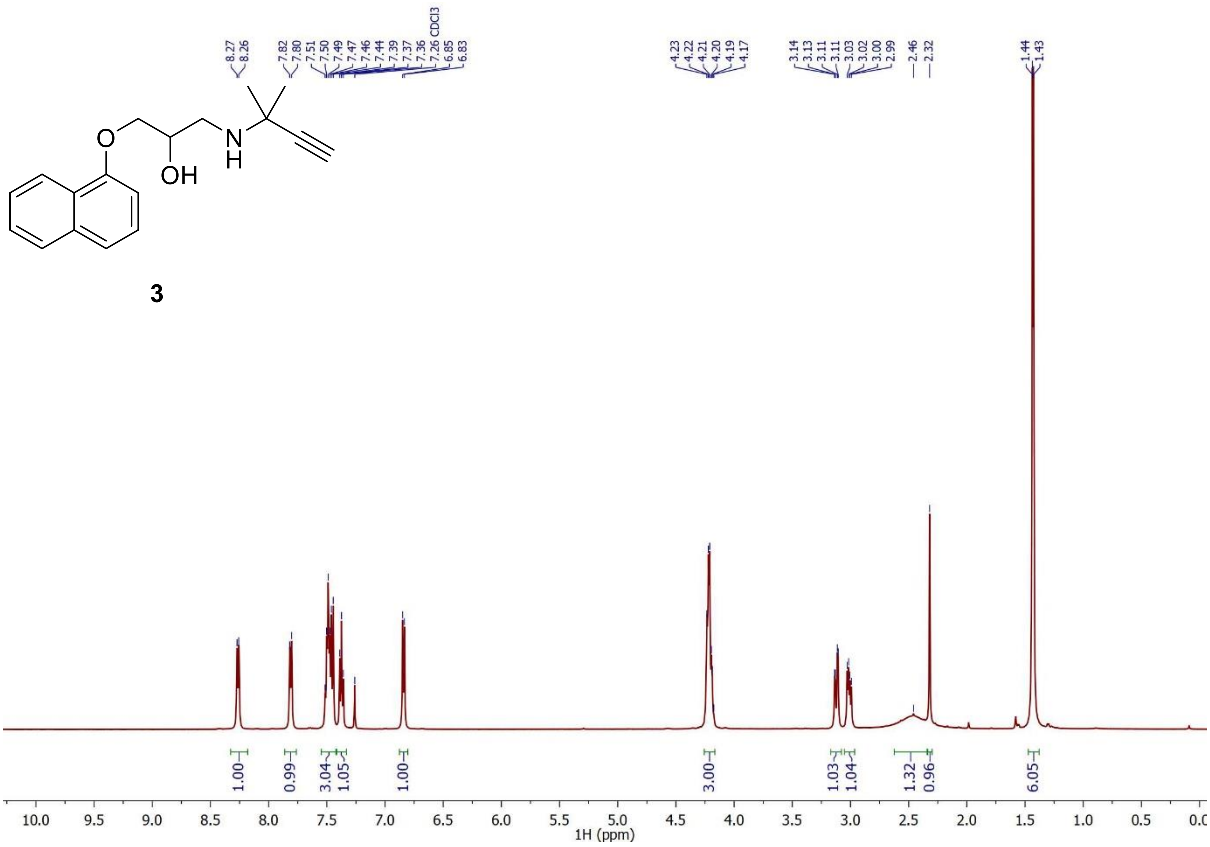

^{13}C NMR of 3

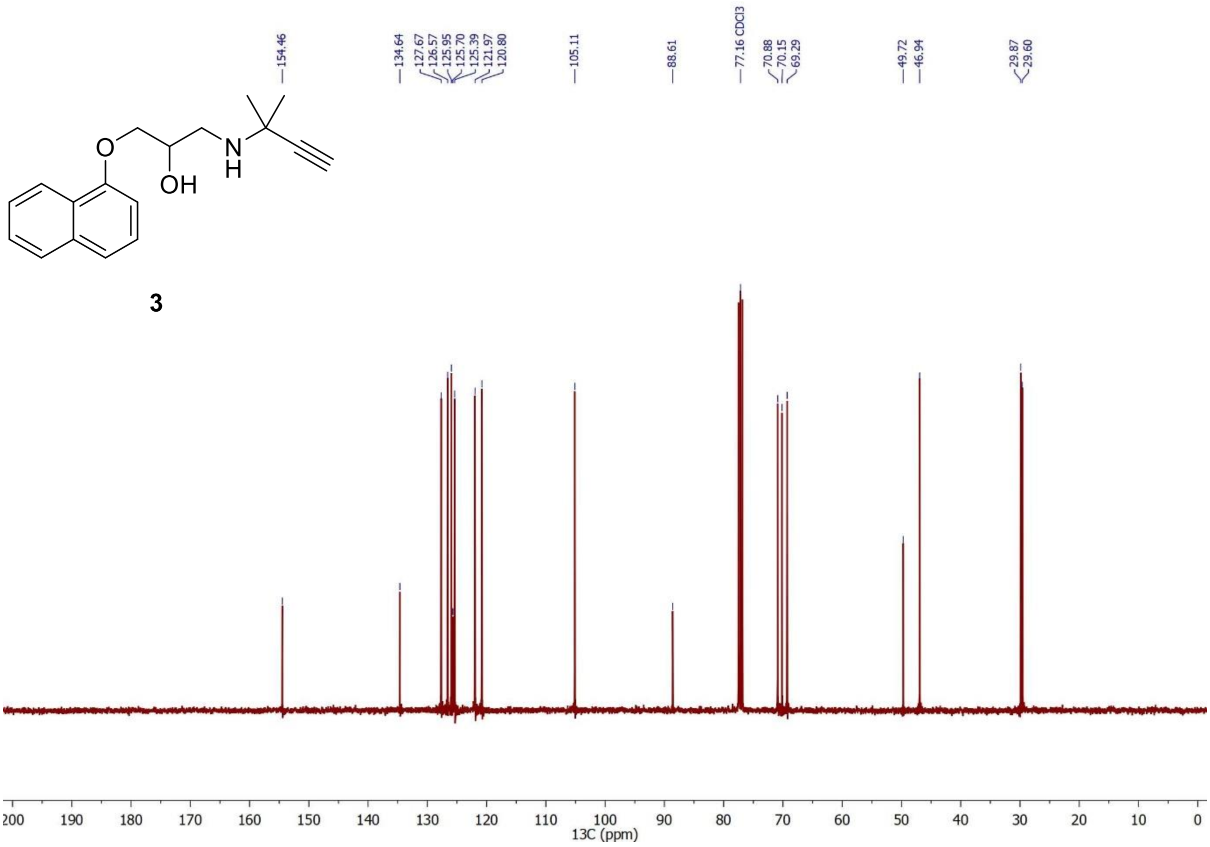

^{1}H NMR of 4

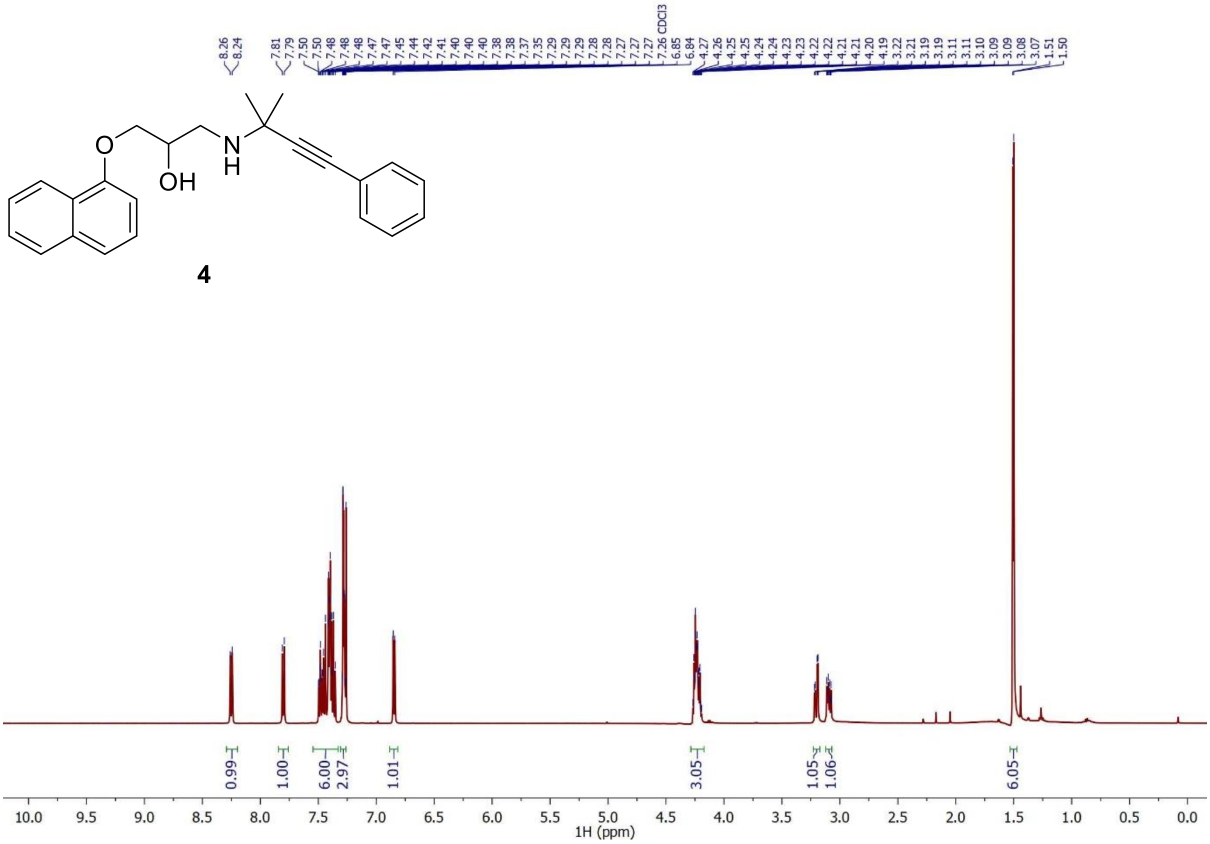

^{13}C NMR of 4

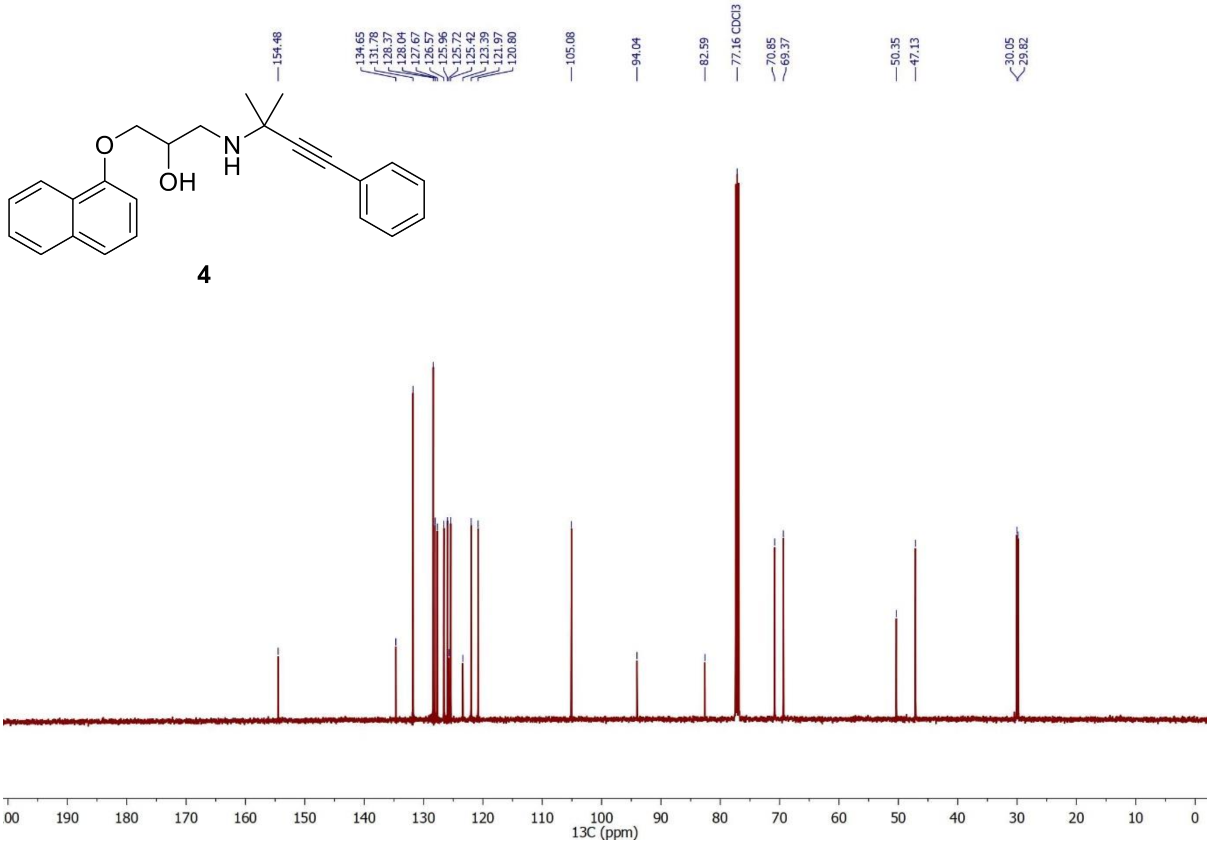

Supplement: Supplementary file 1 — ac4c01285_si_001.pdf [file ac4c01285_si_001.pdf]
